# Supplementary material for: The compact genome of Giardia muris reveals important steps in the evolution of intestinal protozoan parasites
Source: Microb Genom. 2020 Jul 3;6(8):mgen000402. doi: 10.1099/mgen.0.000402 (PMC7641422; doi:10.1099/mgen.0.000402)
Supplement: Supplementary material 1 [file mgen-6-402-s001.pdf]

Supporting information

Figure S1. **A)** *G. muris* cells propagate as trophozoites in the mice small intestine. The cell trophozoites undergo encystation into cysts and are shed in the feces. The cysts are prompted to excyst as they encounter the conditions of the digestive tract. The excyzoites transition into the vegetative trophozoites. Cell material for genomic DNA was harvested from *G. muris* cysts from infected mice. **B)** Sub-telomeric regions in *G. muris*. Sequence motifs in the 10 unitigs terminating in $(TAGGG)^n$ telomeric sequences (purple). Ribosomal DNA sequences (black). Satellite-like repeated sequences (green).

Figure S2. Chromosome-scale synteny between *G. muris* and *G. intestinalis* WB. *G. muris* chromosomes are color coded. The links between the two genomes representing sequence similarity identified by MUMmer promer[96], are colored after the *G. muris* chromosomal color. Circular plot was drawn with circlize[94].

Figure S3. Sequence logo around start codon (A) and stop codon (B) in *G. muris*. C) *G. muris* gene expression from *in vivo* trophozoites. Genes showing top-ranked FPKM ratios (log2 (Trophs/Cysts)) between *in vivo* derived trophozoites and cysts (cut-off value ≥4). The value is indicated in the teal circle. An orange dot indicates that the ortholog of this gene is upregulated (>2 fold) in *G. intestinalis* at 7 hrs, 12 hrs or 22 hrs into encystation [40].

Figure S4. *Trans*- and *cis*-introns in *G. muris*. A) Aligned *trans*- and *cis*-splice junctions in *G. muris.* **B)** Splice-site associated motifs in *trans*-spliced exons. Base-pairing stems are underlined. Bases in red denote the predicted cleavage motif. **C)** Consensus alignment of the cleavage motifs in *G. muris*. **D-E)** Predicted secondary structures of **D)** *cis*-spliced and **E)** *trans*-spliced genes. The secondary structure was predicted using the mfold webserver using default settings and manually curated. GU-rich stretches and cleavage motifs are boxed.

Figure S5. A) Conservation of the VSP C-terminal in *G. muris* and *G. intestinalis.* Sequence logos of conserved motifs in the C-terminal of VSPs in *G. muris* (n=25) (upper panel) and *G. intestinalis* WB (n=183) (lower panel) were generated using MEME v5.0.5. Transmembrane and cytosolic pentapeptides were predicted by the Phobius webserver. **B) VSP and ψVSP evolutionary dynamics.** The ψVSP arrays (defined as >3 ψVSP genes arrayed) in the *G. muris* genome arranged by chromosome, termini arranged to the right. Arrays of ψVSPs without chromosomal context are shown below as orphan arrays. **C)** VSP cluster mostly together in a phylogeny whereas most ψVSP gene arrays show relaxed clustering. Intact VSP genes are colored red, while ψVSP genes are colored according to their resident ψVSP array in panel A. Genes with black label are not found in arrays. **D) Expression measurements of VSP and ψVSP in *G. muris.*** VSP and ψVSP RNA-seq read counts (FPKM) from trophozoites, cysts and excyzoites. Size of the circle corresponds to expression level.

Figure S6. A) Phylogenetic tree of *G. muris* and *G. intestinalis* NEKs. B) Phylogenetic tree of *G. muris* and *G. intestinalis* ARPs. C) Network analysis of *G. muris* and *G. intestinalis* NEKs and ARPs. *G. muris* genes are represented in circle whereas *G. intestinalis* genes are shown in triangle. Blue indicates NEK and pink as ARP. Edges are weighted and scaled by reciprocal BLAST scores with evalue 1e-05 as the cutoff.

Figure S7. Phylogenetic tree of C1 family cysteine proteases in diplomonads. Black: *G. muris*, Red: *S. salmonicida*, Cyan: *G. intestinalis* GS/M, Green: *G. intestinalis* P15, Purple: *G. intestinalis* WB.

Figure S8. Phylogenetic analyses of HGT genes. A) Fructokinase. **B)** Mannose-6-phosphate isomerase. **C)** Transcriptional regulator/sugar kinase. **D)** Arginase. **E)** 2,5-diketo-D-gluconic acid reductase 1. **F)** 2,5-diketo-D-gluconic acid reductase 2. **G)** Carboxymuconolactone decarboxylase. **H)** Ketosteroid isomerase. **I)** Tryptophanase. **J)** Tae4. **K)** Quorum-quenching N-acyl-homoserine lactonase.

Table S1. A) One-to-one ortholog list between *G. muris* and *G. intestinalis* WB. Orthologs inferred by OrthoMCL. **B)** RNA-Seq expression values in excel file. Sorted according to FPKM values. **C)** VSP and ψVSP gene metadata. '-' in the pentapeptide motif represents lack of the 3' end sequence. **D)** List of ventral disc proteins in *G. muris*. **E)** Proteases detected in *G. muris* and *G. intestinalis*. **F)** Detailed information about hits of unique genes in *G. muris.*

Methods S1. Additional methods section

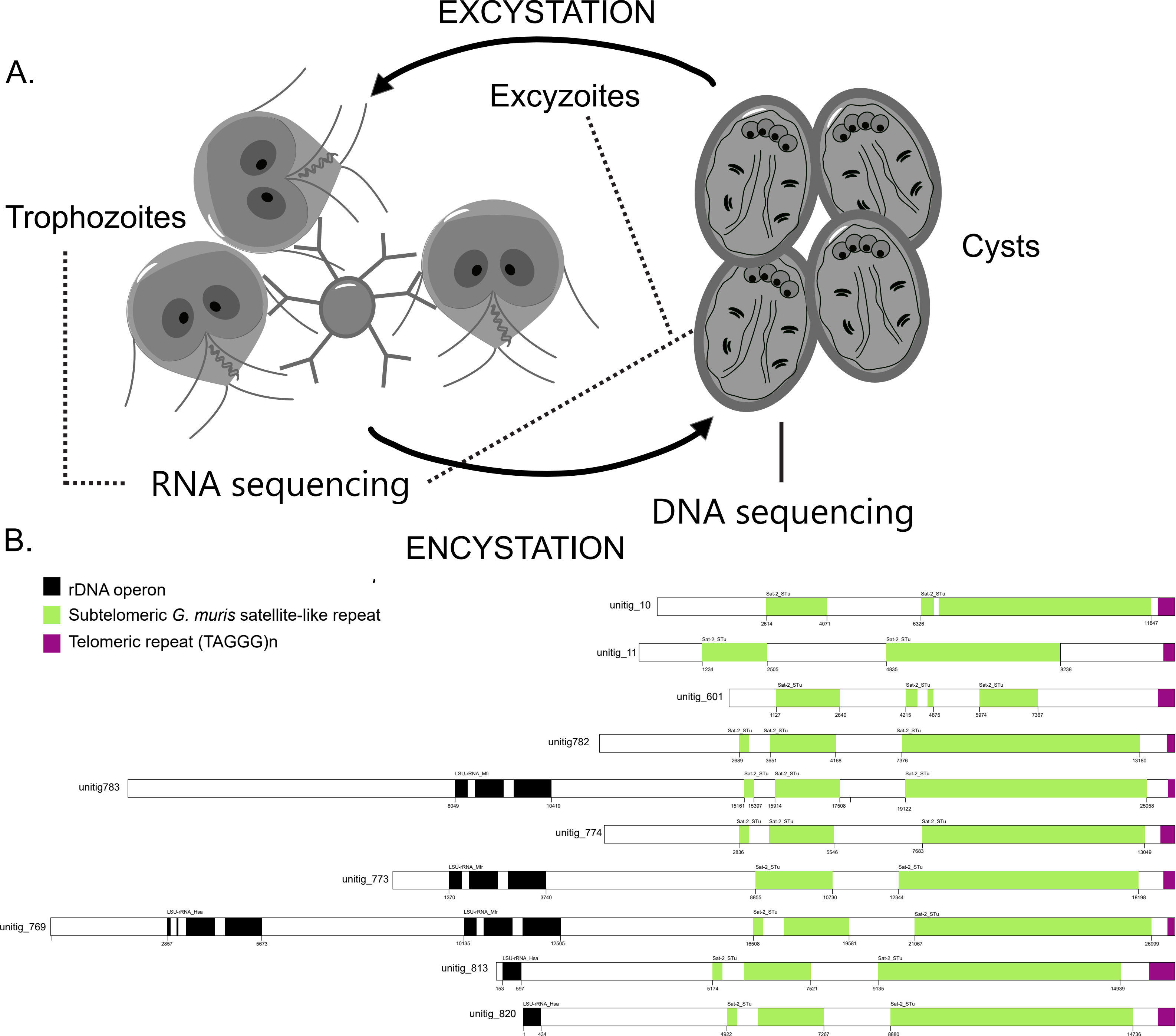

A.
EXCYSTATION
Excyzoites
Trophozoites
Cysts
RNA sequencing
DNA sequencing
ENCYSTATION
B.
rDNA operon
Subtelomeric G. muris satellite-like repeat
Telomeric repeat (TAGGG)n
unitig_10
unitig_11
unitig_601
unitig782
unitig783
unitig_774
unitig_773
unitig_769
unitig_813
unitig_820
Sat-2_STu
LSU-rRNA_Mfr
LSU-rRNA_Hsa

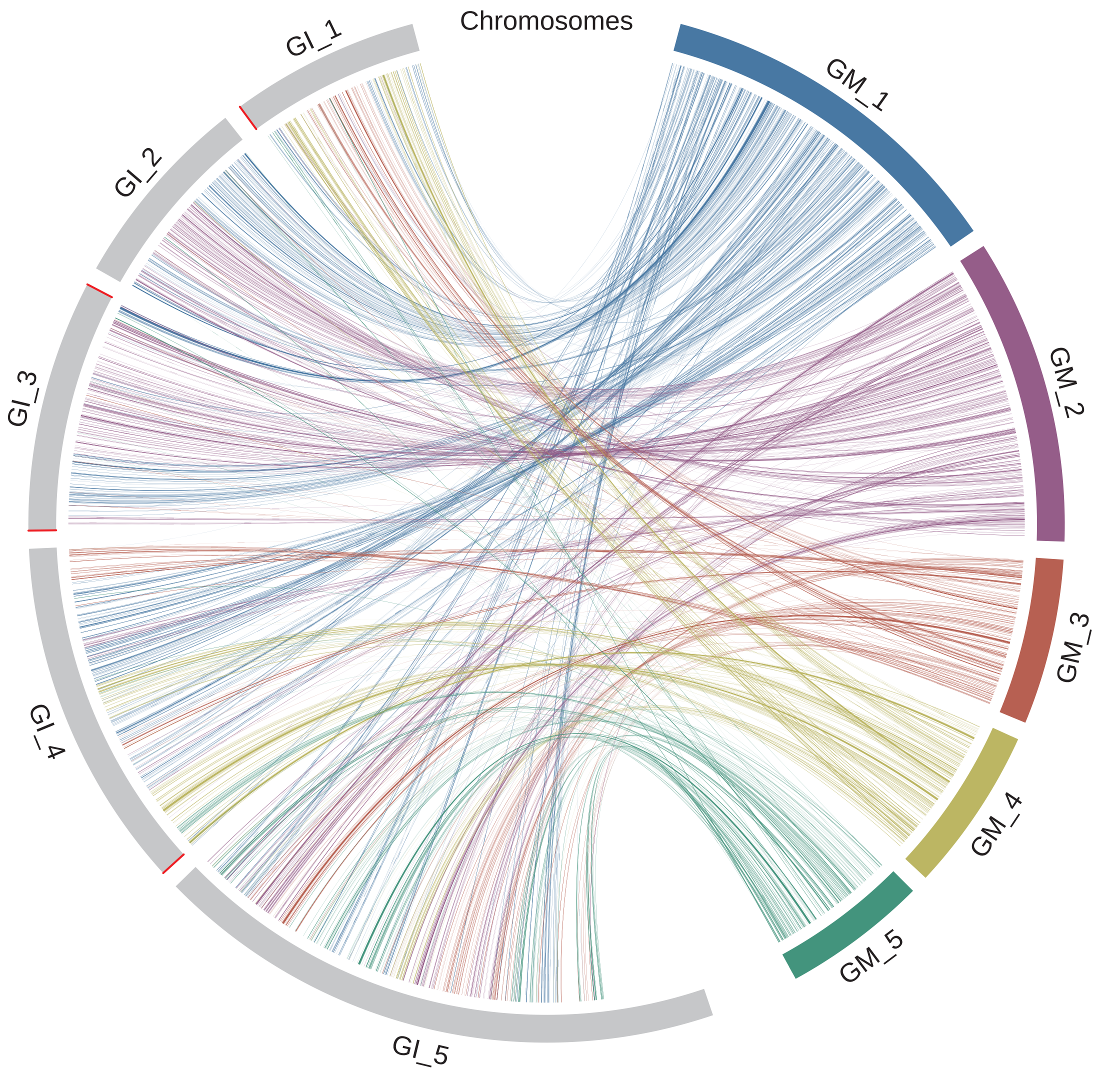
Chromosomes
GI_1
GI_2
GI_3
GI_4
GI_5
GM_1
GM_2
GM_3
GM_4
GM_5

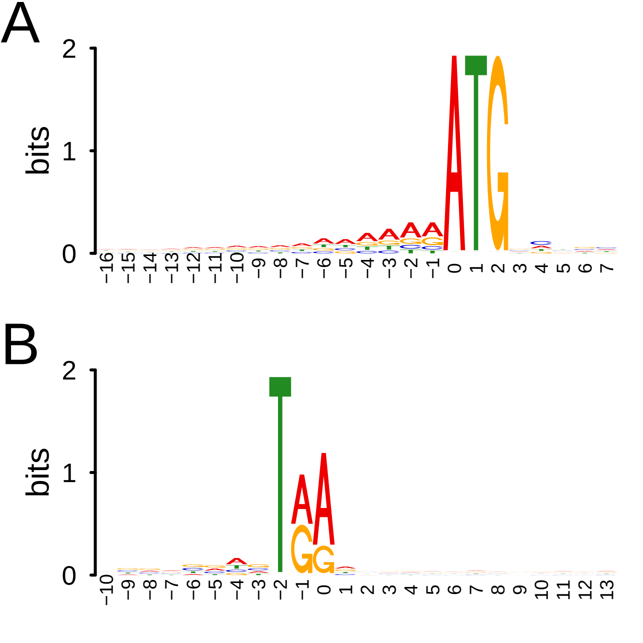
A
B
bits
0
1
2
−16 −15 −14 −13 −12 −11 −10 −9 −8 −7 −6 −5 −4 −3 −2 −1 0 1 2 3 4 5 6 7
−10 −9 −8 −7 −6 −5 −4 −3 −2 −1 0 1 2 3 4 5 6 7 8 9 10 11 12 13

C

LOG2 Trophs/Cysts	Encyst up 90 min	Encyst up 7h	Encyst up 22h	Gene id
9				GMRT_13982
9		●	●	GMRT_13204
9		●	●	GMRT_10774
9			●	GMRT_12407
8				GMRT_13942
8			●	GMRT_13341
8	●	●	●	GMRT_13400
8		●	●	GMRT_16219
8			●	GMRT_13851
8			●	GMRT_10589
8			●	GMRT_15202
8			●	GMRT_14302
7				GMRT_14494
7		●	●	GMRT_11770
7			●	GMRT_12514
7			●	GMRT_13776
7				GMRT_12207
7		●	●	GMRT_10157
7			●	GMRT_12519
7	●			GMRT_10224
7				GMRT_15543
7				GMRT_15329
7		●	●	GMRT_15984
7				GMRT_10478
7				GMRT_13283
7			●	GMRT_13422
7			●	GMRT_11433
6				GMRT_13315
6			●	GMRT_12667
6				GMRT_12476
6				GMRT_15303
6				GMRT_13901
6	●	●		GMRT_12279
6				GMRT_12528
6				GMRT_15601
6				GMRT_15514
6				GMRT_13605
6	●			GMRT_14361
6				GMRT_14093
6				GMRT_10233
6		●	●	GMRT_10574
6				GMRT_14982
6			●	GMRT_15986
6				GMRT_12453
6			●	GMRT_12189
6			●	GMRT_10312
6				GMRT_12190
6				GMRT_14841
6			●	GMRT_12243
6				GMRT_16336
6			●	GMRT_10833
6		●		GMRT_14208
6				GMRT_11693
6		●	●	GMRT_11117
6			●	GMRT_14265
6			●	GMRT_14586
6				GMRT_10367
6			●	GMRT_11957
6				GMRT_12759
6			●	GMRT_15885
6				GMRT_11905
6			●	GMRT_15547
6	●			GMRT_10756
6			●	GMRT_13994
5				GMRT_10377
5	●		●	GMRT_10123
5			●	GMRT_13780
5		●	●	GMRT_15811
5			●	GMRT_14189
5				GMRT_14883
5			●	GMRT_14997
5				GMRT_12394
5				GMRT_10848
5				GMRT_12419
5				GMRT_10135
5				GMRT_15872
5			●	GMRT_16332
5				GMRT_14187
5				GMRT_11145
5				GMRT_12776
5				GMRT_11843
5				GMRT_10541
5				GMRT_15517
5				GMRT_10471
5			●	GMRT_15257
5				GMRT_10501
5				GMRT_12607
5			●	GMRT_14295
5				GMRT_11315
5				GMRT_12559
5				GMRT_13415
5				GMRT_16213
5	●			GMRT_13109
5				GMRT_10567
5			●	GMRT_14575
5				GMRT_12715
5			●	GMRT_15985
5				GMRT_13841
5			●	GMRT_10038
5			●	GMRT_11441
5				GMRT_13609
5			●	GMRT_11004
5		●	●	GMRT_14079
5				GMRT_11698
5		●		GMRT_12381
5			●	GMRT_10030
5	●			GMRT_10585
5			●	GMRT_15240
5				GMRT_14576
5			●	GMRT_10991
5			●	GMRT_10607
5			●	GMRT_11520
5				GMRT_10127
5				GMRT_15829
5		●	●	GMRT_14224
5	●	●	●	GMRT_13401
5				GMRT_12332
5				GMRT_13227
5				GMRT_14385
5			●	GMRT_10590
5			●	GMRT_10989
5				GMRT_12531
5		●	●	GMRT_11758
5				GMRT_15162
5				GMRT_10361
5				GMRT_13783
5	●			GMRT_13387
5			●	GMRT_14264
5			●	GMRT_12874
5			●	GMRT_11847
4				GMRT_13488
4				GMRT_13010
4			●	GMRT_16375
4				GMRT_14214
4				GMRT_12291
4				GMRT_12942
4			●	GMRT_13845
4			●	GMRT_13363
4				GMRT_10998
4				GMRT_13929
4				GMRT_16145
4				GMRT_16374
4				GMRT_12385
4			●	GMRT_14301
4				GMRT_14584
4			●	GMRT_11013
4			●	GMRT_10770
4			●	GMRT_13180
4	●			GMRT_15317
4				GMRT_10811
4				GMRT_12821
4				GMRT_12091
4				GMRT_11966
4			●	GMRT_12130
4				GMRT_13902
4				GMRT_16379
4				GMRT_14952
4				GMRT_13245
4	●			GMRT_15524
4				GMRT_15258
4				GMRT_12300
4				GMRT_16311
4				GMRT_15163
4			●	GMRT_12285
4				GMRT_14430
4			●	GMRT_13601
4	●	●		GMRT_12672
4			●	GMRT_15899
4			●	GMRT_15546
4	●			GMRT_13611
4			●	GMRT_14303
4				GMRT_10495
4				GMRT_15159
4		●		GMRT_10188
4				GMRT_11987
4				GMRT_14267
4	●			GMRT_13101
4				GMRT_16305
4				GMRT_12824
4				GMRT_10401
4	●		●	GMRT_14423
4				GMRT_10296
4	●	●	●	GMRT_11676
4				GMRT_13096
4			●	GMRT_13390
4			●	GMRT_15222
4				GMRT_13985
4				GMRT_12620
4	●			GMRT_10467
4		●	●	GMRT_15068
4				GMRT_12343
4		●	●	GMRT_13794
4			●	GMRT_12815
4		●		GMRT_13570
4				GMRT_11953
4				GMRT_14849
4				GMRT_12799
4				GMRT_13282
4				GMRT_10492

A.

Trans-spliced introns

```
GMRT_12388 GTgtatgtgtg.../ /...ggactaacacgcagCT
GMRT_11576 AGgtatgtccg.../ /...tgactaacacgcagCA
GMRT_10332 GGgtatgtggt.../ /...ttactaacacgcagCT
GMRT_11888 TGatatgttgt.../ /...tcactaacacgcagAC
GMRT_22684 TGgtatggccc.../ /...ttactaacacgcagTC
             .****..               ************
```

Cis-spliced introns

```
GMRT_fx013 GCgtatgtatc    tc    catctaacacgtagAA
GMRT_15227 GGgtatgtgcc (124nt) tcactaacgcgcagAT
GMRT_11066 CCgtatgttca  tatcg   tctctaacacgcagCT
             ******.             .*****.**.**
```

B.

```
GMRT_12388-1     gtatgtgtgtgcgtcagccgctgtgtgttgtgtgtttcctttactcaatgacggtctc

GMRT_12388-1d    gtatgtgtgtgcgtcagccgctgtgtgttgtgtgtttcctttacttaaatggttc

GMRT_12388-2     aaaaatcagcggctgacgcgcaggaccatctggactaacacgcag


GMRT_11576-1     gtatgtccgctgtgtgcgagcatgtgtgttgtgtgtctcctttactcaacttccgcgg

GMRT_11576-1d    gtatgtccgctgtgtgcgagcatgtgtgttgtgtgtctcctttactcaatatcaaattctccattgtatttcat

GMRT_11576-2     aaaaaacctcaagtactggagggaagaaaagctcgcaccagcagaatcttgactaacacgcag

GMRT_11576-2-gt  gtatgtggtgttccgcttgctgtgtgttgtgtgttccttcacttaagttctgtc

GMRT_11576-3     aaaaagagacacgaggacgatcggcaagcggaacacacagtcctttactaacacgcag


GMRT_11888-1     atatgttgtgcgtttcaccccgttgtgtgttgtgtgttcctttttctcaagcattggct

GMRT_11888-2     aaaacggggtgaacgtgcagtcctcactaacacgcag


GMRT_22684-1     gtatggcccctcctccttcactcaagccttct

GMRT_22684-2     aaaggagggaccaatcttactaacacgcag
```

C.

Cleavage motif

```
tcctttactcaa
tcctttacttaa
tcctttactcaa
tccttcacttaa
tcctttttctcaa
tccttcactcaa
*****..**.**
```

D.

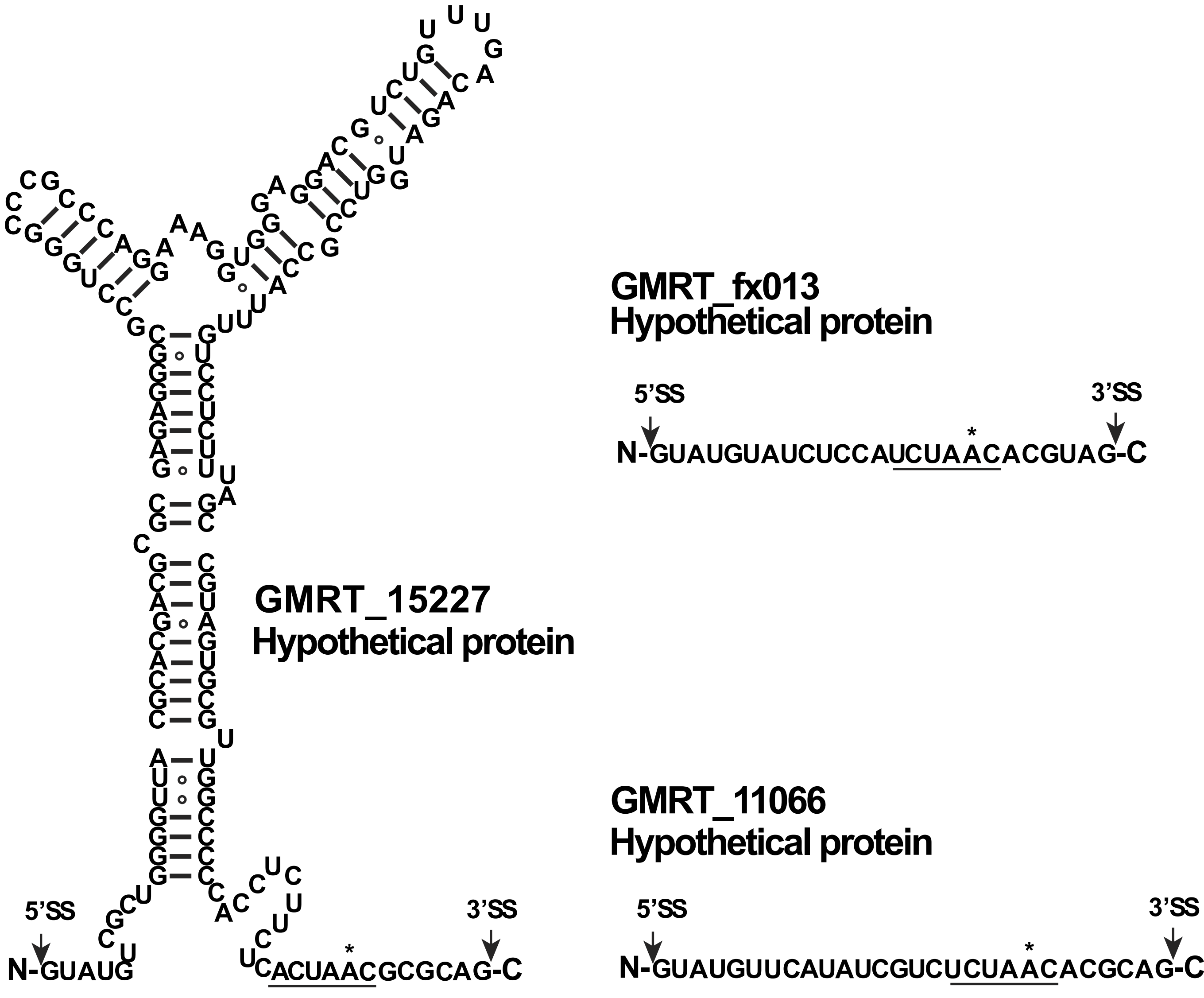
GMRT_fx013
Hypothetical protein
5'SS
3'SS
N-GUAUGUAUCUCCAUCUAACACGUAG-C
GMRT_15227
Hypothetical protein
5'SS
3'SS
GMRT_11066
Hypothetical protein
5'SS
3'SS
N-GUAUGUUCAUAUCGUCUCUAACACGCAG-C

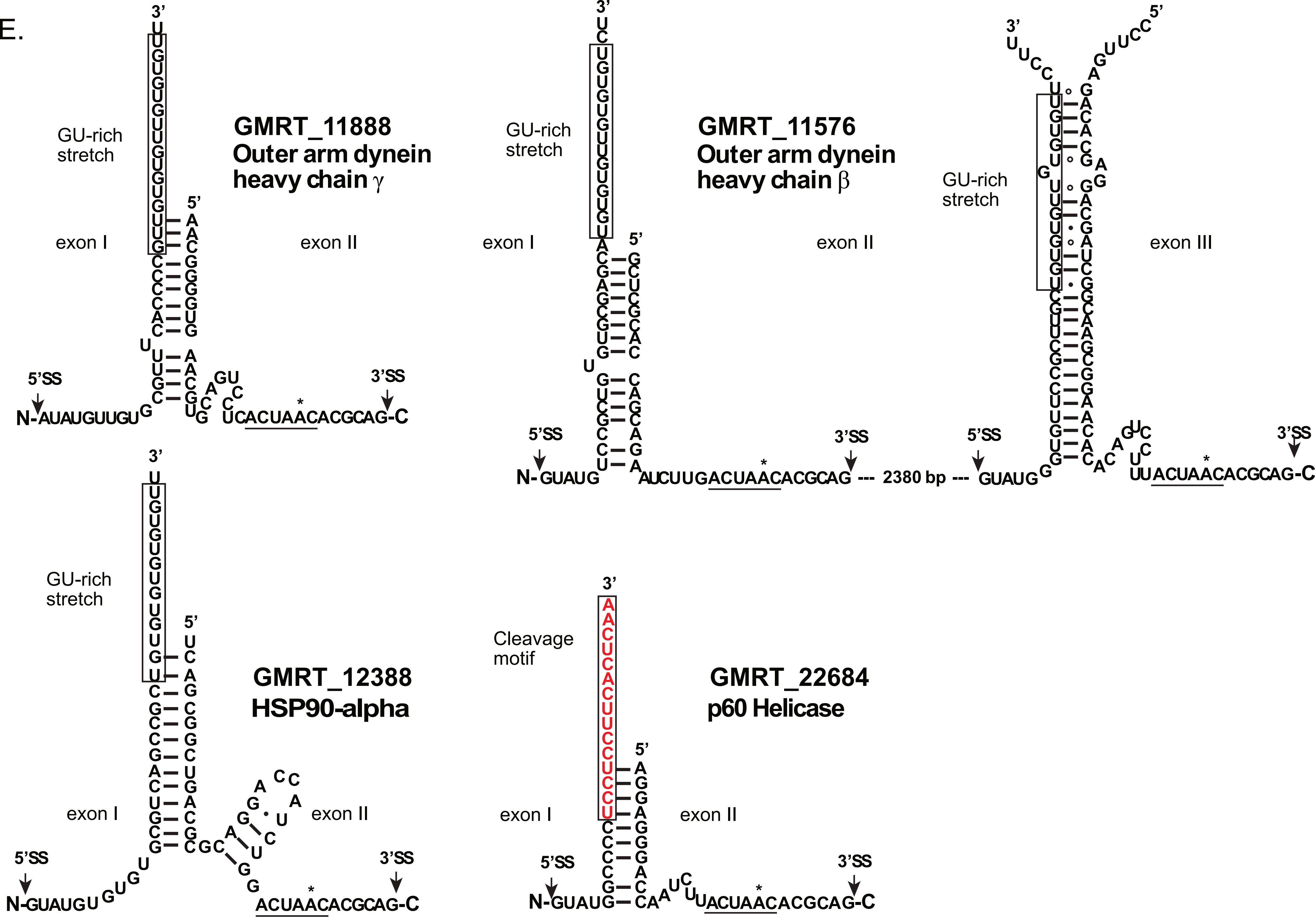
E.
GMRT_11888
Outer arm dynein
heavy chain γ
GU-rich
stretch
exon I
exon II
5'SS
3'SS
N-AUAUGUUGU
UCACUAACACGCAG-C
GMRT_11576
Outer arm dynein
heavy chain β
GU-rich
stretch
exon I
exon II
exon III
5'SS
3'SS
N-GUAUG
AUCUUGACUAACACGCAG --- 2380 bp --- GUAUG
UUACUAACACGCAG-C
GMRT_12388
HSP90-alpha
GU-rich
stretch
exon I
exon II
5'SS
3'SS
N-GUAUGU
ACUAACACGCAG-C
GMRT_22684
p60 Helicase
Cleavage
motif
exon I
exon II
5'SS
3'SS
N-GUAUG
UACUAACACGCAG-C

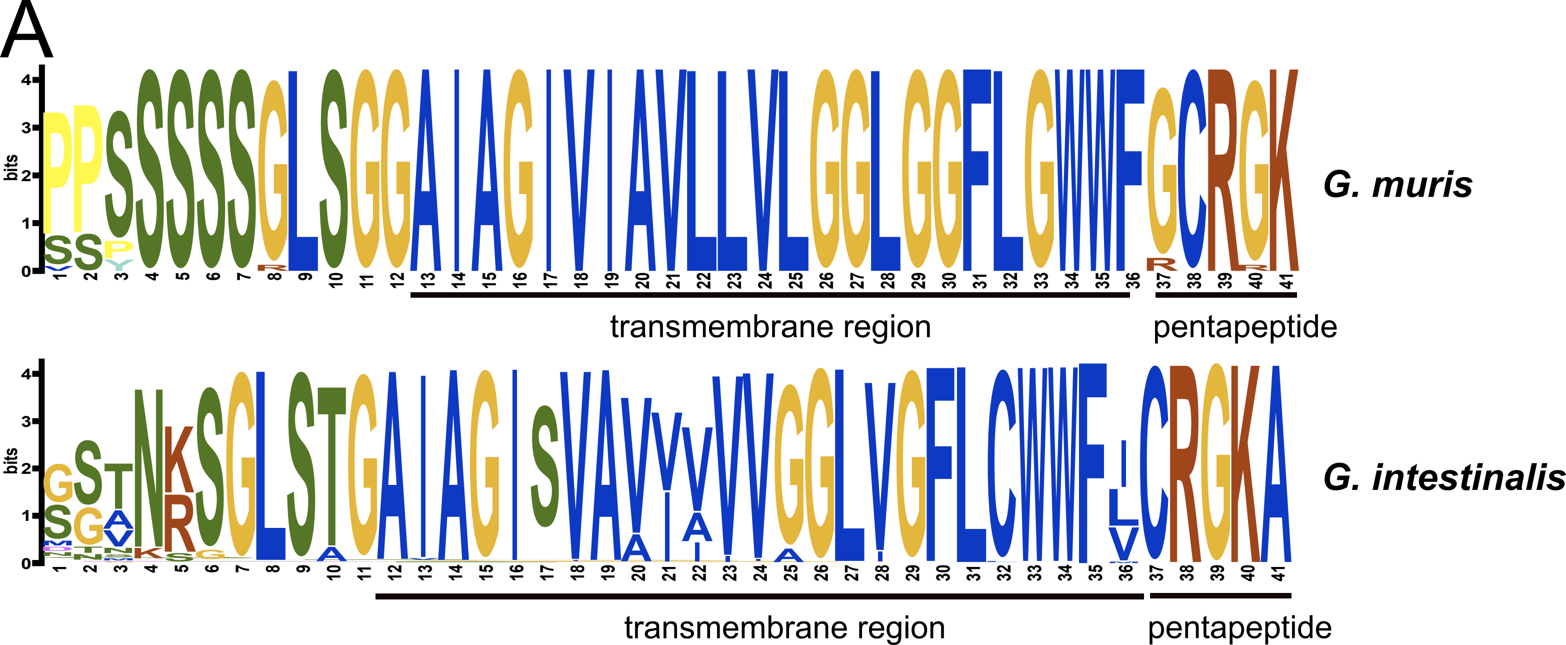
A
bits
G. muris
transmembrane region
pentapeptide
bits
G. intestinalis
transmembrane region
pentapeptide

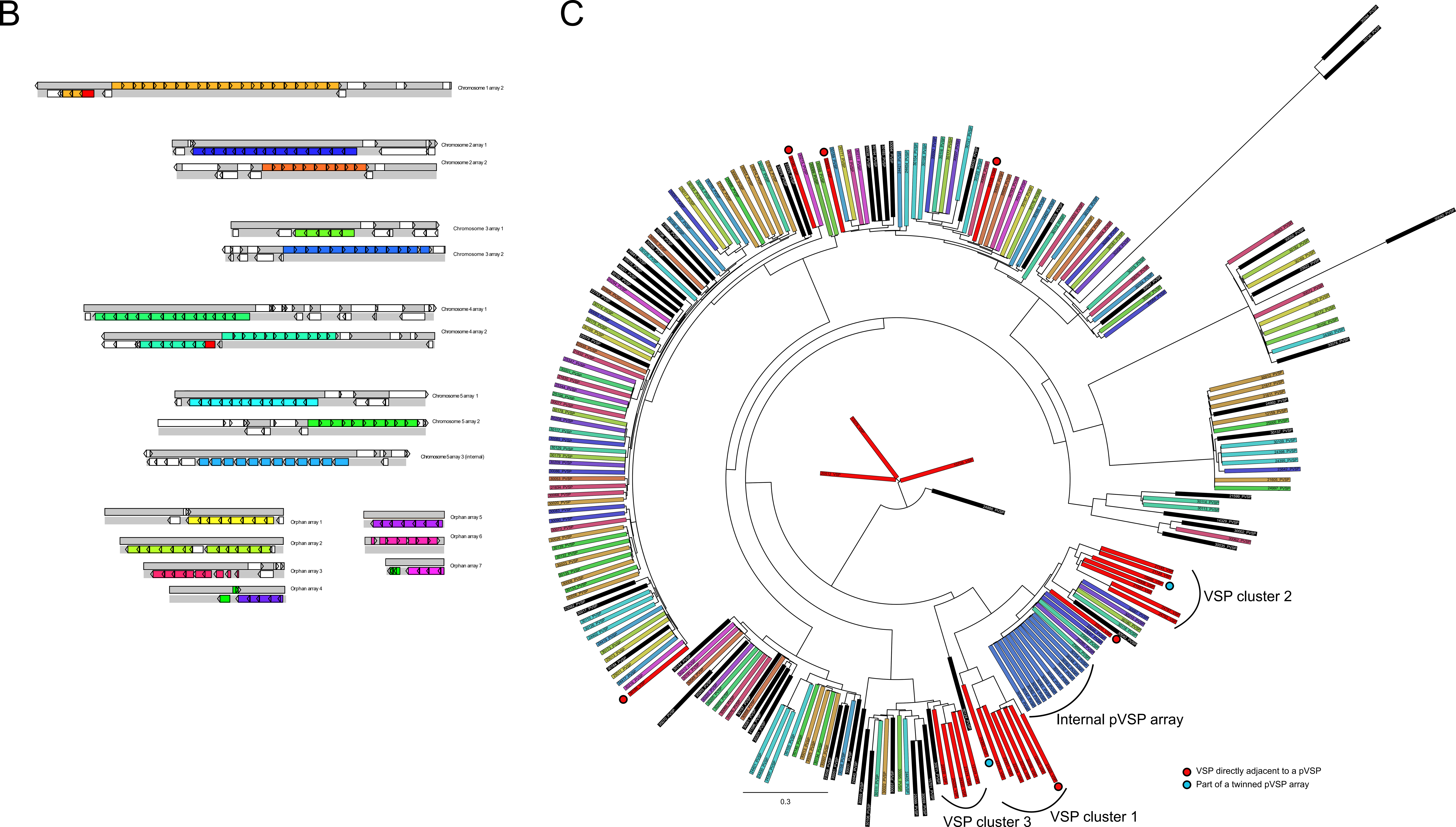
B
Chromosome 1 array 2
Chromosome 2 array 1
Chromosome 2 array 2
Chromosome 3 array 1
Chromosome 3 array 2
Chromosome 4 array 1
Chromosome 4 array 2
Chromosome 5 array 1
Chromosome 5 array 2
Chromosome 5 array 3 (internal)
Orphan array 1
Orphan array 2
Orphan array 3
Orphan array 4
Orphan array 5
Orphan array 6
Orphan array 7
C
VSP cluster 2
Internal pVSP array
VSP cluster 3
VSP cluster 1
0.3
VSP directly adjacent to a pVSP
Part of a twinned pVSP array

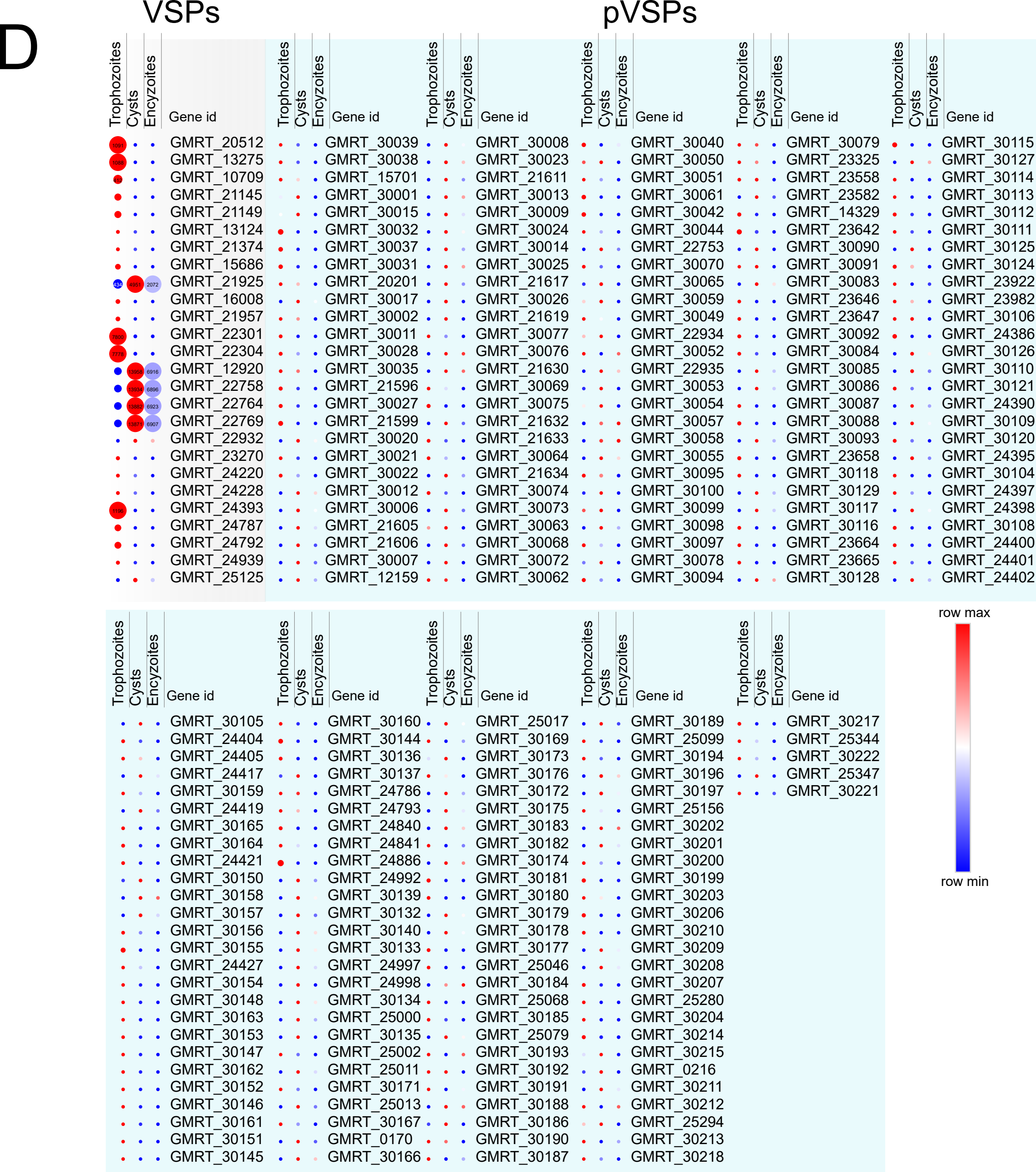
D
VSPs
pVSPs
Trophozoites
Cysts
Encyzoites
Gene id
GMRT_20512
GMRT_13275
GMRT_10709
GMRT_21145
GMRT_21149
GMRT_13124
GMRT_21374
GMRT_15686
GMRT_21925
GMRT_16008
GMRT_21957
GMRT_22301
GMRT_22304
GMRT_12920
GMRT_22758
GMRT_22764
GMRT_22769
GMRT_22932
GMRT_23270
GMRT_24220
GMRT_24228
GMRT_24393
GMRT_24787
GMRT_24792
GMRT_24939
GMRT_25125
GMRT_30039
GMRT_30038
GMRT_15701
GMRT_30001
GMRT_30015
GMRT_30032
GMRT_30037
GMRT_30031
GMRT_20201
GMRT_30017
GMRT_30002
GMRT_30011
GMRT_30028
GMRT_30035
GMRT_21596
GMRT_30027
GMRT_21599
GMRT_30020
GMRT_30021
GMRT_30022
GMRT_30012
GMRT_30006
GMRT_21605
GMRT_21606
GMRT_30007
GMRT_12159
GMRT_30008
GMRT_30023
GMRT_21611
GMRT_30013
GMRT_30009
GMRT_30024
GMRT_30014
GMRT_30025
GMRT_21617
GMRT_30026
GMRT_21619
GMRT_30077
GMRT_30076
GMRT_21630
GMRT_30069
GMRT_30075
GMRT_21632
GMRT_21633
GMRT_30064
GMRT_21634
GMRT_30074
GMRT_30073
GMRT_30063
GMRT_30068
GMRT_30072
GMRT_30062
GMRT_30040
GMRT_30050
GMRT_30051
GMRT_30061
GMRT_30042
GMRT_30044
GMRT_22753
GMRT_30070
GMRT_30065
GMRT_30059
GMRT_30049
GMRT_22934
GMRT_30052
GMRT_22935
GMRT_30053
GMRT_30054
GMRT_30057
GMRT_30058
GMRT_30055
GMRT_30095
GMRT_30100
GMRT_30099
GMRT_30098
GMRT_30097
GMRT_30078
GMRT_30094
GMRT_30079
GMRT_23325
GMRT_23558
GMRT_23582
GMRT_14329
GMRT_23642
GMRT_30090
GMRT_30091
GMRT_30083
GMRT_23646
GMRT_23647
GMRT_30092
GMRT_30084
GMRT_30085
GMRT_30086
GMRT_30087
GMRT_30088
GMRT_30093
GMRT_23658
GMRT_30118
GMRT_30129
GMRT_30117
GMRT_30116
GMRT_23664
GMRT_23665
GMRT_30128
GMRT_30115
GMRT_30127
GMRT_30114
GMRT_30113
GMRT_30112
GMRT_30111
GMRT_30125
GMRT_30124
GMRT_23922
GMRT_23982
GMRT_30106
GMRT_24386
GMRT_30126
GMRT_30110
GMRT_30121
GMRT_24390
GMRT_30109
GMRT_30120
GMRT_24395
GMRT_30104
GMRT_24397
GMRT_24398
GMRT_30108
GMRT_24400
GMRT_24401
GMRT_24402
GMRT_30105
GMRT_24404
GMRT_24405
GMRT_24417
GMRT_30159
GMRT_24419
GMRT_30165
GMRT_30164
GMRT_24421
GMRT_30150
GMRT_30158
GMRT_30157
GMRT_30156
GMRT_30155
GMRT_24427
GMRT_30154
GMRT_30148
GMRT_30163
GMRT_30153
GMRT_30147
GMRT_30162
GMRT_30152
GMRT_30146
GMRT_30161
GMRT_30151
GMRT_30145
GMRT_30160
GMRT_30144
GMRT_30136
GMRT_30137
GMRT_24786
GMRT_24793
GMRT_24840
GMRT_24841
GMRT_24886
GMRT_24992
GMRT_30139
GMRT_30132
GMRT_30140
GMRT_30133
GMRT_24997
GMRT_24998
GMRT_30134
GMRT_25000
GMRT_30135
GMRT_25002
GMRT_25011
GMRT_30171
GMRT_25013
GMRT_30167
GMRT_0170
GMRT_30166
GMRT_25017
GMRT_30169
GMRT_30173
GMRT_30176
GMRT_30172
GMRT_30175
GMRT_30183
GMRT_30182
GMRT_30174
GMRT_30181
GMRT_30180
GMRT_30179
GMRT_30178
GMRT_30177
GMRT_25046
GMRT_30184
GMRT_25068
GMRT_30185
GMRT_25079
GMRT_30193
GMRT_30192
GMRT_30191
GMRT_30188
GMRT_30186
GMRT_30190
GMRT_30187
GMRT_30189
GMRT_25099
GMRT_30194
GMRT_30196
GMRT_30197
GMRT_25156
GMRT_30202
GMRT_30201
GMRT_30200
GMRT_30199
GMRT_30203
GMRT_30206
GMRT_30210
GMRT_30209
GMRT_30208
GMRT_30207
GMRT_25280
GMRT_30204
GMRT_30214
GMRT_30215
GMRT_0216
GMRT_30211
GMRT_30212
GMRT_25294
GMRT_30213
GMRT_30218
GMRT_30217
GMRT_25344
GMRT_30222
GMRT_25347
GMRT_30221
row max
row min

A

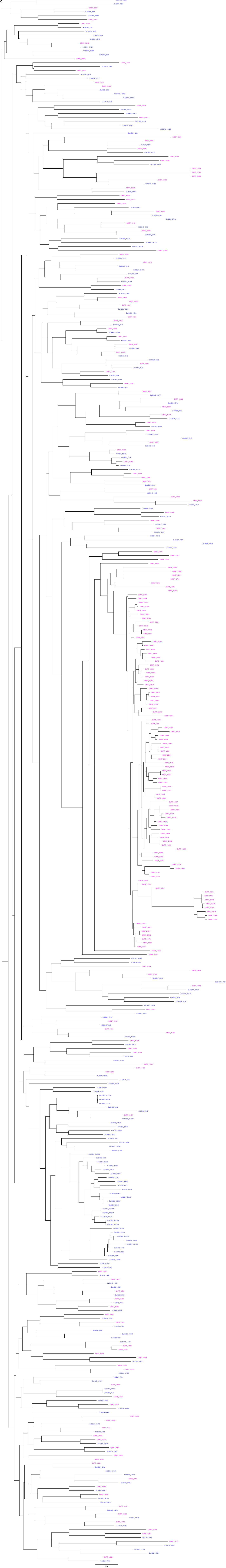

B

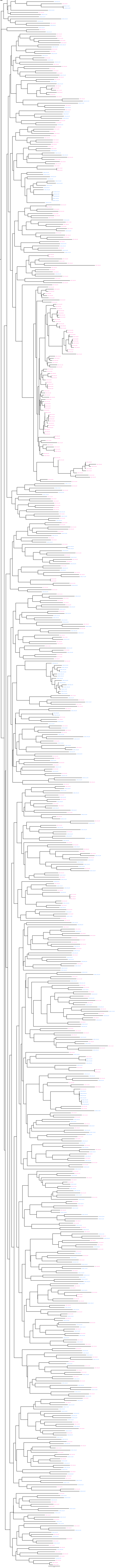

C

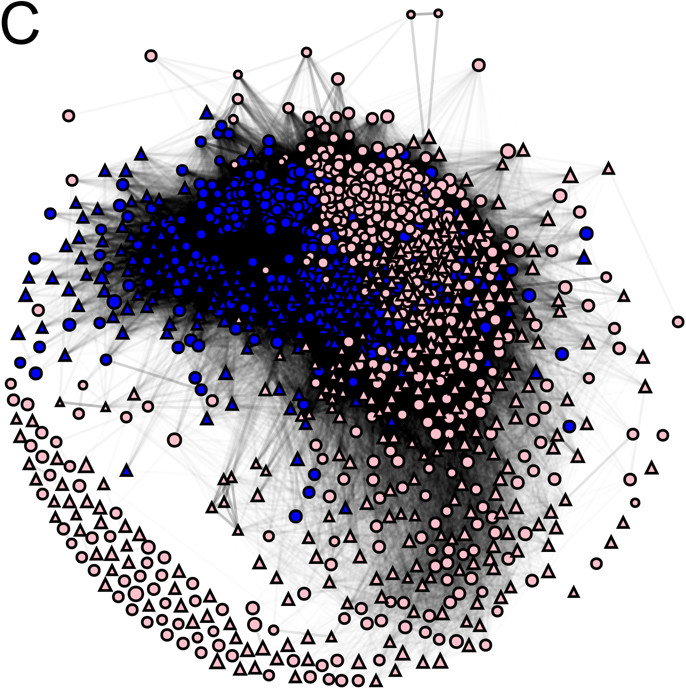

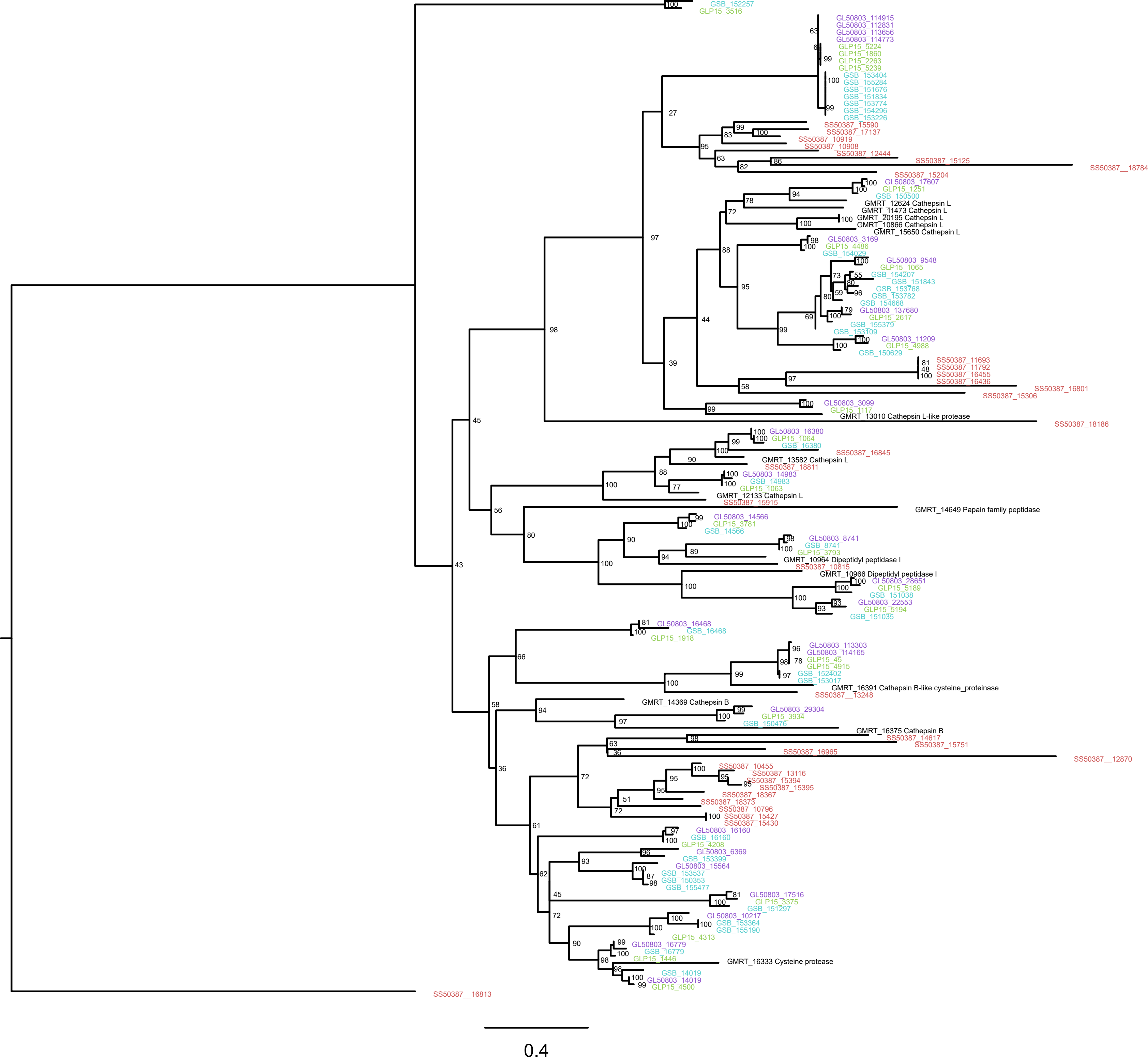
GSB_152257
GLP15_3516
GL50803_114915
GL50803_112831
GL50803_113656
GL50803_114773
GLP15_5224
GLP15_1860
GLP15_2263
GLP15_5239
GSB_153404
GSB_155284
GSB_151676
GSB_151834
GSB_153774
GSB_154296
GSB_153226
SS50387_15590
SS50387_17137
SS50387_10919
SS50387_10908
SS50387_12444
SS50387_15125
SS50387__18784
SS50387_15204
GL50803_17607
GLP15_1251
GSB_150500
GMRT_12624 Cathepsin L
GMRT_11473 Cathepsin L
GMRT_20195 Cathepsin L
GMRT_10866 Cathepsin L
GMRT_15650 Cathepsin L
GL50803_3169
GLP15_4486
GSB_154029
GL50803_9548
GLP15_1065
GSB_154207
GSB_151843
GSB_153768
GSB_153782
GSB_154668
GL50803_137680
GLP15_2617
GSB_155379
GSB_153109
GL50803_11209
GLP15_4988
GSB_150629
SS50387_11693
SS50387_11792
SS50387_16455
SS50387_16436
SS50387_16801
SS50387_15306
GL50803_3099
GLP15_1117
GMRT_13010 Cathepsin L-like protease
SS50387_18186
GL50803_16380
GLP15_1064
GSB_16380
SS50387_16845
GMRT_13582 Cathepsin L
SS50387_18811
GL50803_14983
GSB_14983
GLP15_1063
GMRT_12133 Cathepsin L
SS50387_15915
GMRT_14649 Papain family peptidase
GL50803_14566
GLP15_3781
GSB_14566
GL50803_8741
GSB_8741
GLP15_3793
GMRT_10964 Dipeptidyl peptidase I
SS50387_10815
GMRT_10966 Dipeptidyl peptidase I
GL50803_28651
GLP15_5189
GSB_151038
GL50803_22553
GLP15_5194
GSB_151035
GL50803_16468
GSB_16468
GLP15_1918
GL50803_113303
GL50803_114165
GLP15_45
GLP15_4915
GSB_152402
GSB_153017
GMRT_16391 Cathepsin B-like cysteine_proteinase
SS50387_13248
GMRT_14369 Cathepsin B
GL50803_29304
GLP15_3934
GSB_150476
GMRT_16375 Cathepsin B
SS50387_14617
SS50387_15751
SS50387_16965
SS50387__12870
SS50387_10455
SS50387_13116
SS50387_15394
SS50387_15395
SS50387_18367
SS50387_18373
SS50387_10796
SS50387_15427
SS50387_15430
GL50803_16160
GSB_16160
GLP15_4208
GL50803_6369
GSB_153399
GL50803_15564
GSB_153537
GSB_150353
GSB_155477
GL50803_17516
GLP15_3375
GSB_151297
GL50803_10217
GSB_153364
GSB_155190
GLP15_4313
GL50803_16779
GSB_16779
GLP15_1446
GMRT_16333 Cysteine protease
GSB_14019
GL50803_14019
GLP15_4500
SS50387__16813
0.4

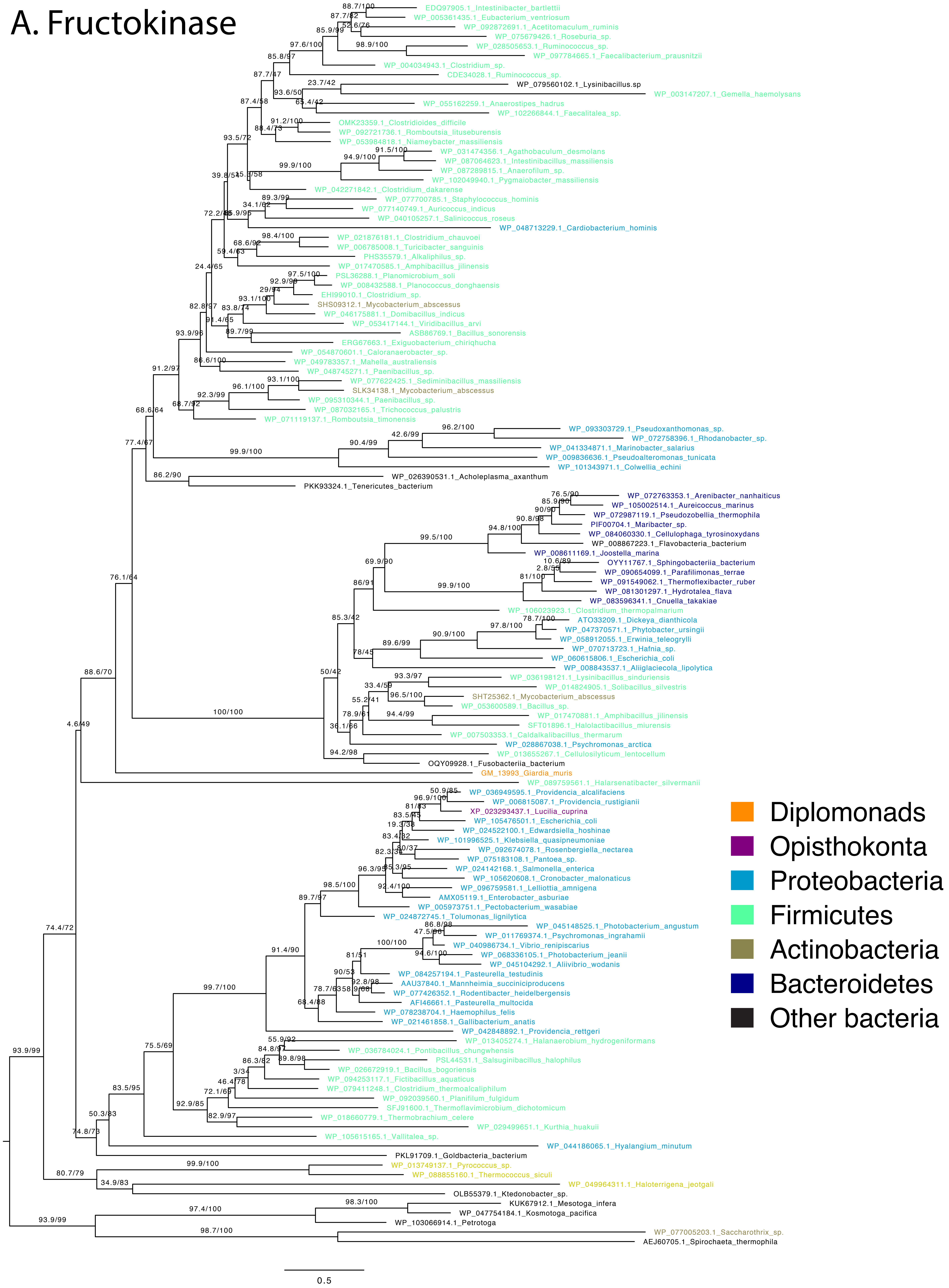
A. Fructokinase
EDQ97905.1_Intestinibacter_bartlettii
WP_005361435.1_Eubacterium_ventriosum
WP_092872691.1_Acetitomaculum_ruminis
WP_075679426.1_Roseburia_sp.
WP_028505653.1_Ruminococcus_sp.
WP_097784665.1_Faecalibacterium_prausnitzii
WP_004034943.1_Clostridium_sp.
CDE34028.1_Ruminococcus_sp.
WP_079560102.1_Lysinibacillus.sp
WP_003147207.1_Gemella_haemolysans
WP_055162259.1_Anaerostipes_hadrus
WP_102268844.1_Faecalitalea_sp.
OMK23359.1_Clostridioides_difficile
WP_092721736.1_Romboutsia_lituseburensis
WP_053984818.1_Niameybacter_massiliensis
WP_031474356.1_Agathobaculum_desmolans
WP_087064623.1_Intestinibacillus_massiliensis
WP_087289815.1_Anaerofilum_sp.
WP_102049940.1_Pygmaiobacter_massiliensis
WP_042271842.1_Clostridium_dakarense
WP_077700785.1_Staphylococcus_hominis
WP_077140749.1_Auricoccus_indicus
WP_040105257.1_Salinicoccus_roseus
WP_048713229.1_Cardiobacterium_hominis
WP_021876181.1_Clostridium_chauvoei
WP_006785008.1_Turicibacter_sanguinis
PHS35579.1_Alkaliphilus_sp.
WP_017470585.1_Amphibacillus_jilinensis
PSL36288.1_Planomicrobium_soli
WP_008432588.1_Planococcus_donghaensis
EHI99010.1_Clostridium_sp.
SHS09312.1_Mycobacterium_abscessus
WP_046175881.1_Domibacillus_indicus
WP_053417144.1_Viridibacillus_arvi
ASB86769.1_Bacillus_sonorensis
ERG67663.1_Exiguobacterium_chiriqhucha
WP_054870601.1_Caloranaerobacter_sp.
WP_049783357.1_Mahella_australiensis
WP_048745271.1_Paenibacillus_sp.
WP_077622425.1_Sediminibacillus_massiliensis
SLK34138.1_Mycobacterium_abscessus
WP_095310344.1_Paenibacillus_sp.
WP_087032165.1_Trichococcus_palustris
WP_071119137.1_Romboutsia_timonensis
WP_093303729.1_Pseudoxanthomonas_sp.
WP_072758396.1_Rhodanobacter_sp.
WP_041334871.1_Marinobacter_salarius
WP_009836636.1_Pseudoalteromonas_tunicata
WP_101343971.1_Colwellia_echini
WP_026390531.1_Acholeplasma_axanthum
PKK93324.1_Tenericutes_bacterium
WP_072763353.1_Arenibacter_nanhaiticus
WP_105002514.1_Aureicoccus_marinus
WP_072987119.1_Pseudozobellia_thermophila
PIF00704.1_Maribacter_sp.
WP_084060330.1_Cellulophaga_tyrosinoxydans
WP_008867223.1_Flavobacteria_bacterium
WP_008611169.1_Joostella_marina
OYY11767.1_Sphingobacteriia_bacterium
WP_090654099.1_Parafilimonas_terrae
WP_091549062.1_Thermoflexibacter_ruber
WP_081301297.1_Hydrotalea_flava
WP_083596341.1_Cnuella_takakiae
WP_106023923.1_Clostridium_thermopalmarium
ATO33209.1_Dickeya_dianthicola
WP_047370571.1_Phytobacter_ursingii
WP_058912055.1_Erwinia_teleogrylli
WP_070713723.1_Hafnia_sp.
WP_060615806.1_Escherichia_coli
WP_008843537.1_Aliiglaciecola_lipolytica
WP_036198121.1_Lysinibacillus_sinduriensis
WP_014824905.1_Solibacillus_silvestris
SHT25362.1_Mycobacterium_abscessus
WP_053600589.1_Bacillus_sp.
WP_017470881.1_Amphibacillus_jilinensis
SFT01896.1_Halolactibacillus_miurensis
WP_007503353.1_Caldalkalibacillus_thermarum
WP_028867038.1_Psychromonas_arctica
WP_013655267.1_Cellulosilyticum_lentocellum
OQY09928.1_Fusobacteriia_bacterium
GM_13993_Giardia_muris
WP_089759561.1_Halarsenatibacter_silvermanii
WP_036949595.1_Providencia_alcalifaciens
WP_006815087.1_Providencia_rustigianii
XP_023293437.1_Lucilia_cuprina
WP_105476501.1_Escherichia_coli
WP_024522100.1_Edwardsiella_hoshinae
WP_101996525.1_Klebsiella_quasipneumoniae
WP_092674078.1_Rosenbergiella_nectarea
WP_075183108.1_Pantoea_sp.
WP_024142168.1_Salmonella_enterica
WP_105620608.1_Cronobacter_malonaticus
WP_096759581.1_Lelliottia_amnigena
AMX05119.1_Enterobacter_asburiae
WP_005973751.1_Pectobacterium_wasabiae
WP_024872745.1_Tolumonas_lignilytica
WP_045148525.1_Photobacterium_angustum
WP_011769374.1_Psychromonas_ingrahamii
WP_040986734.1_Vibrio_renipiscarius
WP_068336105.1_Photobacterium_jeanii
WP_045104292.1_Aliivibrio_wodanis
WP_084257194.1_Pasteurella_testudinis
AAU37840.1_Mannheimia_succiniciproducens
WP_077426352.1_Rodentibacter_heidelbergensis
AFI46661.1_Pasteurella_multocida
WP_078238704.1_Haemophilus_felis
WP_021461858.1_Gallibacterium_anatis
WP_042848892.1_Providencia_rettgeri
WP_013405274.1_Halanaerobium_hydrogeniformans
WP_036784024.1_Pontibacillus_chungwhensis
PSL44531.1_Salsuginibacillus_halophilus
WP_026672919.1_Bacillus_bogoriensis
WP_094253117.1_Fictibacillus_aquaticus
WP_079411248.1_Clostridium_thermoalcaliphilum
WP_092039560.1_Planifilum_fulgidum
SFJ91600.1_Thermoflavimicrobium_dichotomicum
WP_018660779.1_Thermobrachium_celere
WP_029499651.1_Kurthia_huakuii
WP_105615165.1_Vallitalea_sp.
WP_044186065.1_Hyalangium_minutum
PKL91709.1_Goldbacteria_bacterium
WP_013749137.1_Pyrococcus_sp.
WP_088855160.1_Thermococcus_siculi
WP_049964311.1_Haloterrigena_jeotgali
OLB55379.1_Ktedonobacter_sp.
KUK67912.1_Mesotoga_infera
WP_047754184.1_Kosmotoga_pacifica
WP_103066914.1_Petrotoga
WP_077005203.1_Saccharothrix_sp.
AEJ60705.1_Spirochaeta_thermophila
Diplomonads
Opisthokonta
Proteobacteria
Firmicutes
Actinobacteria
Bacteroidetes
Other bacteria
0.5

B. ManA

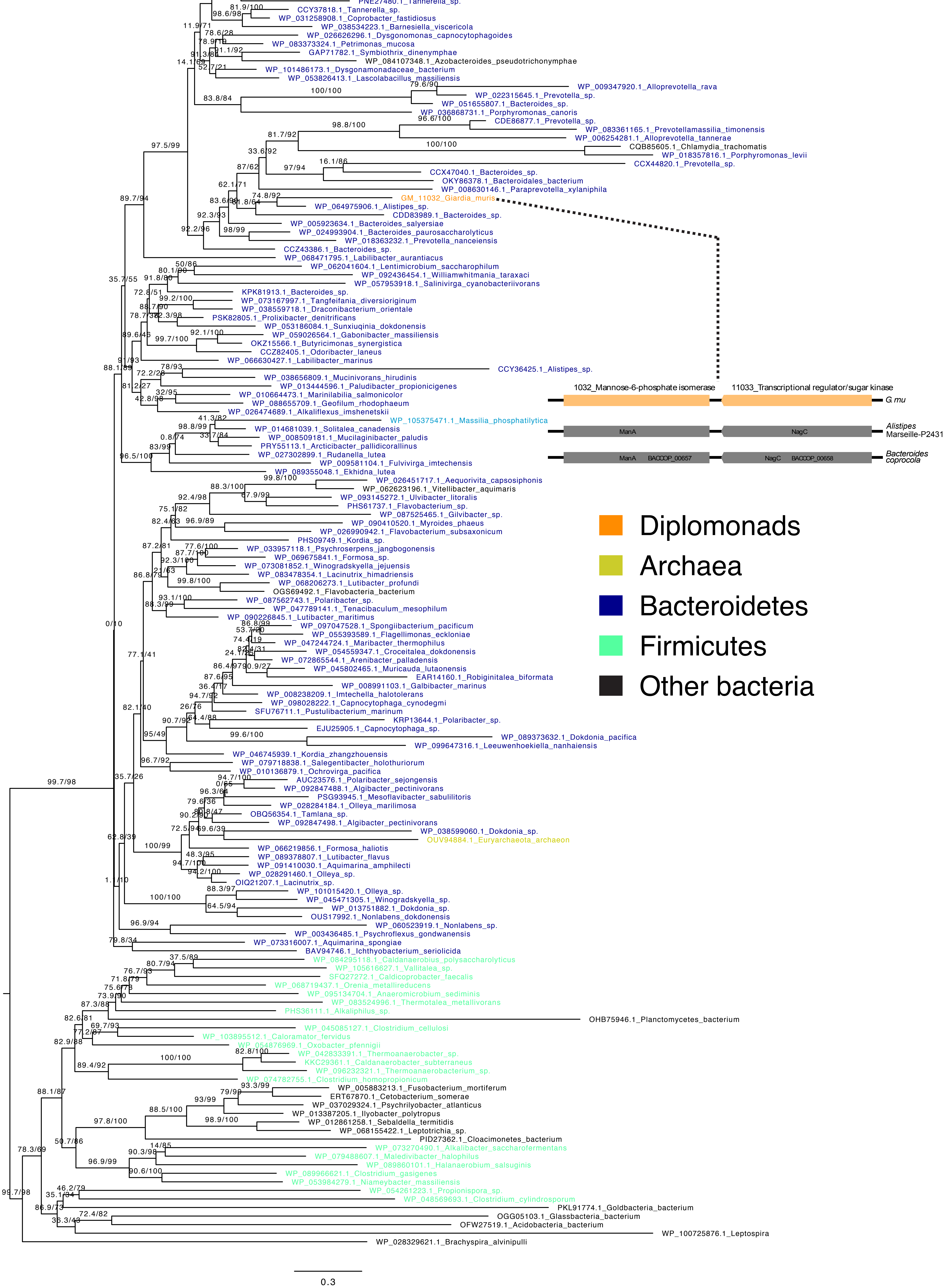

WP_080905946.1_Parabacteroides_sp.
WP_036880967.1_Porphyromonas_sp.
WP_087330492.1_Parabacteroides_sp.
WP_085536262.1_Massilibacteroides_vaginae
PNE27480.1_Tannerella_sp.
CCY37818.1_Tannerella_sp.
WP_031258908.1_Coprobacter_fastidiosus
WP_038534223.1_Barnesiella_viscericola
WP_026626296.1_Dysgonomonas_capnocytophagoides
WP_083373324.1_Petrimonas_mucosa
GAP71782.1_Symbiothrix_dinenymphae
WP_084107348.1_Azobacteroides_pseudotrichonymphae
WP_101486173.1_Dysgonamonadaceae_bacterium
WP_053826413.1_Lascolabacillus_massiliensis
WP_009347920.1_Alloprevotella_rava
WP_022315645.1_Prevotella_sp.
WP_051655807.1_Bacteroides_sp.
WP_036868731.1_Porphyromonas_canoris
CDE86877.1_Prevotella_sp.
WP_083361165.1_Prevotellamassilia_timonensis
WP_006254281.1_Alloprevotella_tannerae
CQB85605.1_Chlamydia_trachomatis
WP_018357816.1_Porphyromonas_levii
CCX44820.1_Prevotella_sp.
CCX47040.1_Bacteroides_sp.
OKY86378.1_Bacteroidales_bacterium
WP_008630146.1_Paraprevotella_xylaniphila
GM_11032_Giardia_muris
WP_064975906.1_Alistipes_sp.
CDD83989.1_Bacteroides_sp.
WP_005923634.1_Bacteroides_salyersiae
WP_024993904.1_Bacteroides_paurosaccharolyticus
WP_018363232.1_Prevotella_nanceiensis
CCZ43386.1_Bacteroides_sp.
WP_068471795.1_Labilibacter_aurantiacus
WP_062041604.1_Lentimicrobium_saccharophilum
WP_092436454.1_Williamwhitmania_taraxaci
WP_057953918.1_Salinivirga_cyanobacteriivorans
KPK81913.1_Bacteroides_sp.
WP_073167997.1_Tangfeifania_diversioriginum
WP_038559718.1_Draconibacterium_orientale
PSK82805.1_Prolixibacter_denitrificans
WP_053186084.1_Sunxiuqinia_dokdonensis
WP_059026564.1_Gabonibacter_massiliensis
OKZ15566.1_Butyricimonas_synergistica
CCZ82405.1_Odoribacter_laneus
WP_066630427.1_Labilibacter_marinus
CCY36425.1_Alistipes_sp.
WP_038656809.1_Mucinivorans_hirudinis
WP_013444596.1_Paludibacter_propionicigenes
WP_010664473.1_Marinilabilia_salmonicolor
WP_088655709.1_Geofilum_rhodophaeum
WP_026474689.1_Alkaliflexus_imshenetskii
WP_105375471.1_Massilia_phosphatilytica
WP_014681039.1_Solitalea_canadensis
WP_008509181.1_Mucilaginibacter_paludis
PRY55113.1_Arcticibacter_pallidicorallinus
WP_027302899.1_Rudanella_lutea
WP_009581104.1_Fulvivirga_imtechensis
WP_089355048.1_Ekhidna_lutea
WP_026451717.1_Aequorivita_capsosiphonis
WP_062623196.1_Vitellibacter_aquimaris
WP_093145272.1_Ulvibacter_litoralis
PHS61737.1_Flavobacterium_sp.
WP_087525465.1_Gilvibacter_sp.
WP_090410520.1_Myroides_phaeus
WP_026990942.1_Flavobacterium_subsaxonicum
PHS09749.1_Kordia_sp.
WP_033957118.1_Psychroserpens_jangbogonensis
WP_069675841.1_Formosa_sp.
WP_073081852.1_Winogradskyella_jejuensis
WP_083478354.1_Lacinutrix_himadriensis
WP_068206273.1_Lutibacter_profundi
OGS69492.1_Flavobacteria_bacterium
WP_087562743.1_Polaribacter_sp.
WP_047789141.1_Tenacibaculum_mesophilum
WP_090226845.1_Lutibacter_maritimus
WP_097047528.1_Spongiibacterium_pacificum
WP_055393589.1_Flagellimonas_eckloniae
WP_047244724.1_Maribacter_thermophilus
WP_054559347.1_Croceitalea_dokdonensis
WP_072865544.1_Arenibacter_palladensis
WP_045802465.1_Muricauda_lutaonensis
EAR14160.1_Robiginitalea_biformata
WP_008991103.1_Galbibacter_marinus
WP_008238209.1_Imtechella_halotolerans
WP_098028222.1_Capnocytophaga_cynodegmi
SFU76711.1_Pustulibacterium_marinum
KRP13644.1_Polaribacter_sp.
EJU25905.1_Capnocytophaga_sp.
WP_089373632.1_Dokdonia_pacifica
WP_099647316.1_Leeuwenhoekiella_nanhaiensis
WP_046745939.1_Kordia_zhangzhouensis
WP_079718838.1_Salegentibacter_holothuriorum
WP_010136879.1_Ochrovirga_pacifica
AUC23576.1_Polaribacter_sejongensis
WP_092847488.1_Algibacter_pectinivorans
PSG93945.1_Mesoflavibacter_sabulilitoris
WP_028284184.1_Olleya_marilimosa
OBQ56354.1_Tamlana_sp.
WP_092847498.1_Algibacter_pectinivorans
WP_038599060.1_Dokdonia_sp.
OUV94884.1_Euryarchaeota_archaeon
WP_066219856.1_Formosa_haliotis
WP_089378807.1_Lutibacter_flavus
WP_091410030.1_Aquimarina_amphilecti
WP_028291460.1_Olleya_sp.
OIQ21207.1_Lacinutrix_sp.
WP_101015420.1_Olleya_sp.
WP_045471305.1_Winogradskyella_sp.
WP_013751882.1_Dokdonia_sp.
OUS17992.1_Nonlabens_dokdonensis
WP_060523919.1_Nonlabens_sp.
WP_003436485.1_Psychroflexus_gondwanensis
WP_073316007.1_Aquimarina_spongiae
BAV94746.1_Ichthyobacterium_seriolicida
WP_084295118.1_Caldanaerobius_polysaccharolyticus
WP_105616627.1_Vallitalea_sp.
SFQ27272.1_Caldicoprobacter_faecalis
WP_068719437.1_Orenia_metallireducens
WP_095134704.1_Anaeromicrobium_sediminis
WP_083524996.1_Thermotalea_metallivorans
PHS36111.1_Alkaliphilus_sp.
OHB75946.1_Planctomycetes_bacterium
WP_045085127.1_Clostridium_cellulosi
WP_103895512.1_Caloramator_fervidus
WP_054876969.1_Oxobacter_pfennigii
WP_042833391.1_Thermoanaerobacter_sp.
KKC29361.1_Caldanaerobacter_subterraneus
WP_096232321.1_Thermoanaerobacterium_sp.
WP_074782755.1_Clostridium_homopropionicum
WP_005883213.1_Fusobacterium_mortiferum
ERT67870.1_Cetobacterium_somerae
WP_037029324.1_Psychrilyobacter_atlanticus
WP_013387205.1_Ilyobacter_polytropus
WP_012861258.1_Sebaldella_termitidis
WP_068155422.1_Leptotrichia_sp.
PID27362.1_Cloacimonetes_bacterium
WP_073270490.1_Alkalibacter_saccharofermentans
WP_079488607.1_Maledivibacter_halophilus
WP_089860101.1_Halanaerobium_salsuginis
WP_089966621.1_Clostridium_gasigenes
WP_053984279.1_Niameybacter_massiliensis
WP_054261223.1_Propionispora_sp.
WP_048569693.1_Clostridium_cylindrosporum
PKL91774.1_Goldbacteria_bacterium
OGG05103.1_Glassbacteria_bacterium
OFW27519.1_Acidobacteria_bacterium
WP_100725876.1_Leptospira
WP_028329621.1_Brachyspira_alvinipulli
0.3
1032_Mannose-6-phosphate isomerase
11033_Transcriptional regulator/sugar kinase
G. mu
ManA
NagC
Alistipes
Marseille-P2431
ManA BACCOP_00657
NagC BACCOP_00658
Bacteroides
coprocola
Diplomonads
Archaea
Bacteroidetes
Firmicutes
Other bacteria

C. Transcriptional regulator

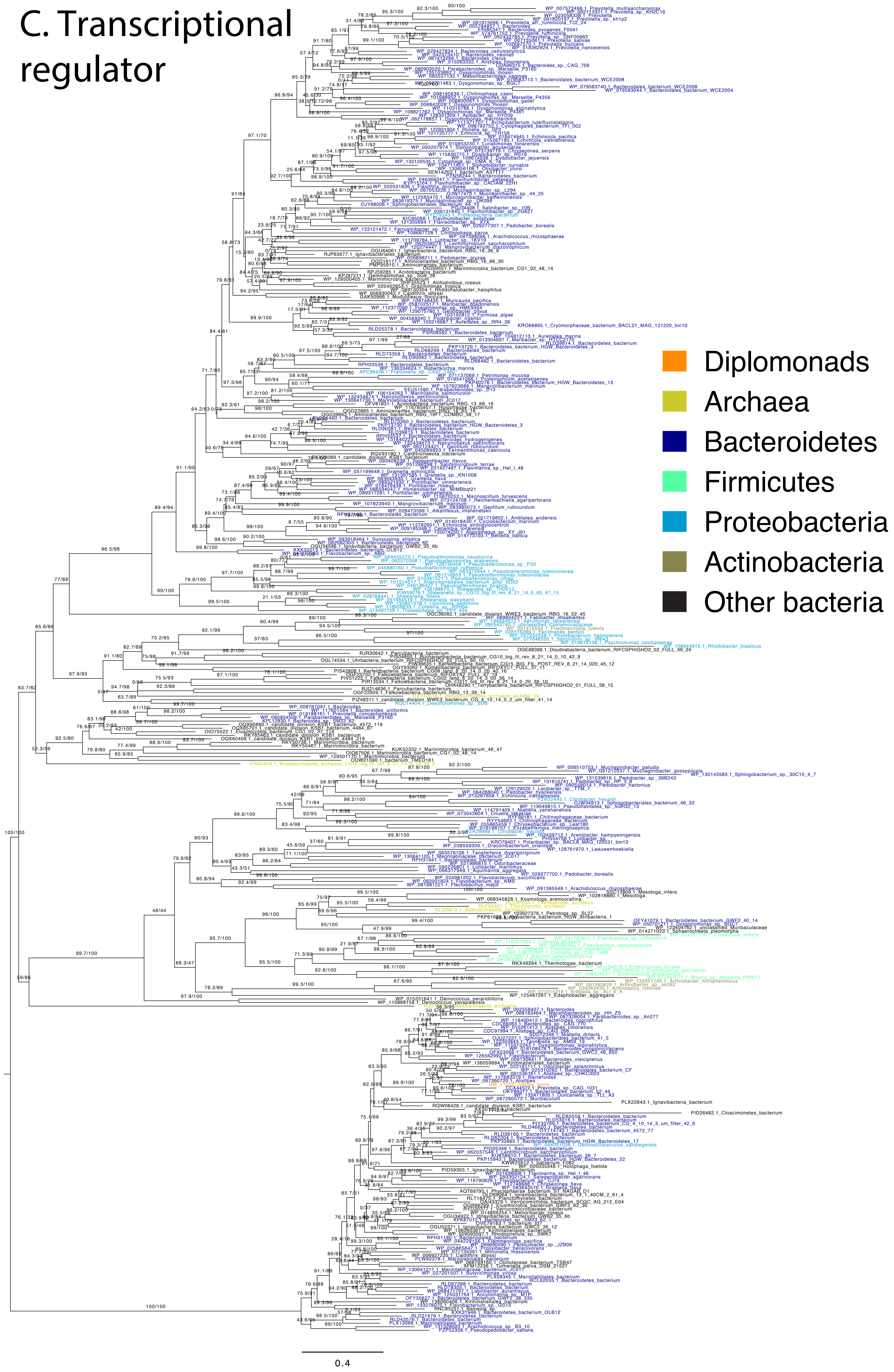

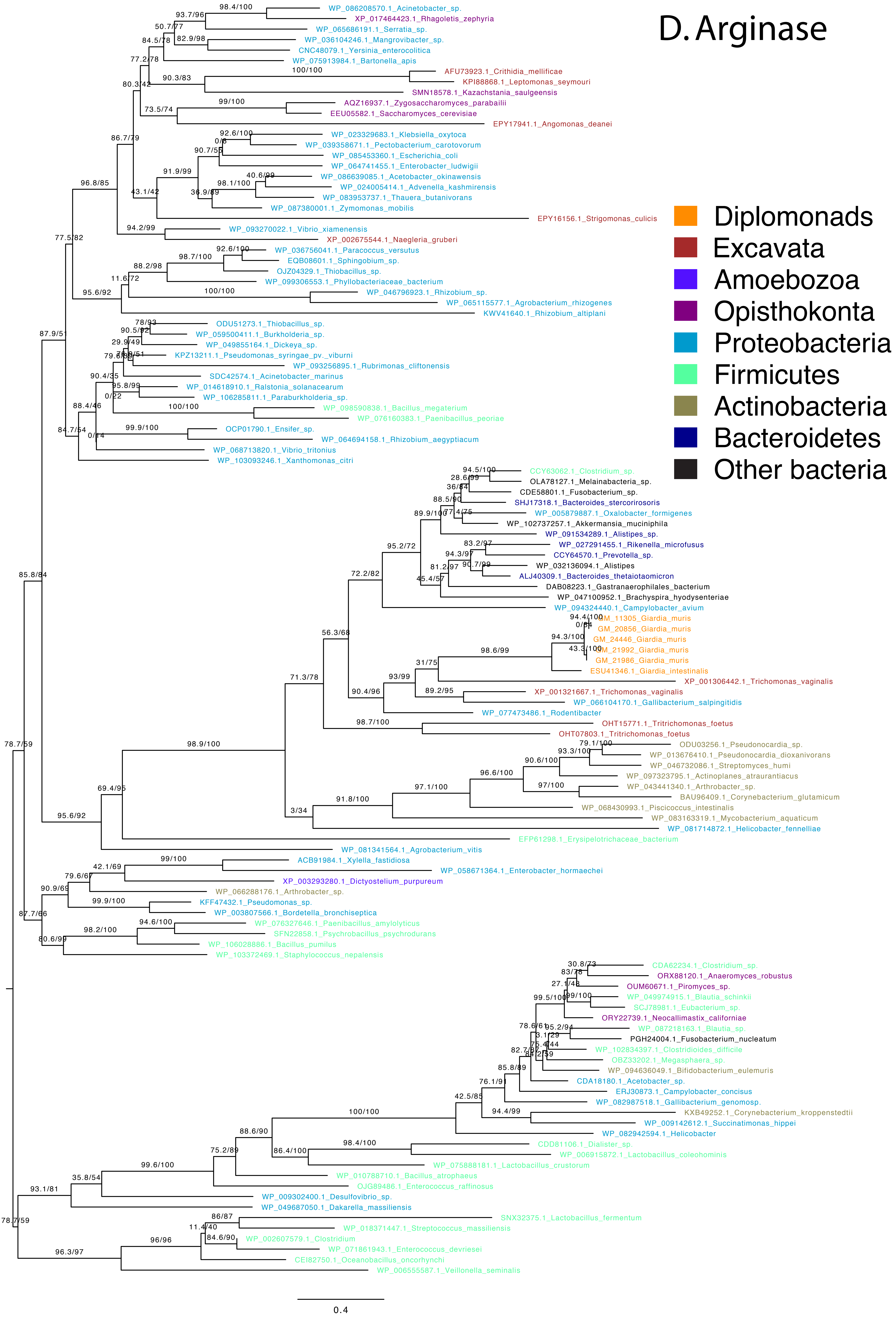
D. Arginase
Diplomonads
Excavata
Amoebozoa
Opisthokonta
Proteobacteria
Firmicutes
Actinobacteria
Bacteroidetes
Other bacteria
WP_086208570.1_Acinetobacter_sp.
XP_017464423.1_Rhagoletis_zephyria
WP_065686191.1_Serratia_sp.
WP_036104246.1_Mangrovibacter_sp.
CNC48079.1_Yersinia_enterocolitica
WP_075913984.1_Bartonella_apis
AFU73923.1_Crithidia_mellificae
KPI88868.1_Leptomonas_seymouri
SMN18578.1_Kazachstania_saulgeensis
AQZ16937.1_Zygosaccharomyces_parabailii
EEU05582.1_Saccharomyces_cerevisiae
EPY17941.1_Angomonas_deanei
WP_023329683.1_Klebsiella_oxytoca
WP_039358671.1_Pectobacterium_carotovorum
WP_085453360.1_Escherichia_coli
WP_064741455.1_Enterobacter_ludwigii
WP_086639085.1_Acetobacter_okinawensis
WP_024005414.1_Advenella_kashmirensis
WP_083953737.1_Thauera_butanivorans
WP_087380001.1_Zymomonas_mobilis
EPY16156.1_Strigomonas_culicis
WP_093270022.1_Vibrio_xiamenensis
XP_002675544.1_Naegleria_gruberi
WP_036756041.1_Paracoccus_versutus
EQB08601.1_Sphingobium_sp.
OJZ04329.1_Thiobacillus_sp.
WP_099306553.1_Phyllobacteriaceae_bacterium
WP_046796923.1_Rhizobium_sp.
WP_065115577.1_Agrobacterium_rhizogenes
KWV41640.1_Rhizobium_altiplani
ODU51273.1_Thiobacillus_sp.
WP_059500411.1_Burkholderia_sp.
WP_049855164.1_Dickeya_sp.
KPZ13211.1_Pseudomonas_syringae_pv._viburni
WP_093256895.1_Rubrimonas_cliftonensis
SDC42574.1_Acinetobacter_marinus
WP_014618910.1_Ralstonia_solanacearum
WP_106285811.1_Paraburkholderia_sp.
WP_098590838.1_Bacillus_megaterium
WP_076160383.1_Paenibacillus_peoriae
OCP01790.1_Ensifer_sp.
WP_064694158.1_Rhizobium_aegyptiacum
WP_068713820.1_Vibrio_tritonius
WP_103093246.1_Xanthomonas_citri
CCY63062.1_Clostridium_sp.
OLA78127.1_Melainabacteria_sp.
CDE58801.1_Fusobacterium_sp.
SHJ17318.1_Bacteroides_stercorirosoris
WP_005879887.1_Oxalobacter_formigenes
WP_102737257.1_Akkermansia_muciniphila
WP_091534289.1_Alistipes_sp.
WP_027291455.1_Rikenella_microfusus
CCY64570.1_Prevotella_sp.
WP_032136094.1_Alistipes
ALJ40309.1_Bacteroides_thetaiotaomicron
DAB08223.1_Gastranaerophilales_bacterium
WP_047100952.1_Brachyspira_hyodysenteriae
WP_094324440.1_Campylobacter_avium
GM_11305_Giardia_muris
GM_20856_Giardia_muris
GM_24446_Giardia_muris
GM_21992_Giardia_muris
GM_21986_Giardia_muris
ESU41346.1_Giardia_intestinalis
XP_001306442.1_Trichomonas_vaginalis
XP_001321667.1_Trichomonas_vaginalis
WP_066104170.1_Gallibacterium_salpingitidis
WP_077473486.1_Rodentibacter
OHT15771.1_Tritrichomonas_foetus
OHT07803.1_Tritrichomonas_foetus
ODU03256.1_Pseudonocardia_sp.
WP_013676410.1_Pseudonocardia_dioxanivorans
WP_046732086.1_Streptomyces_humi
WP_097323795.1_Actinoplanes_atraurantiacus
WP_043441340.1_Arthrobacter_sp.
BAU96409.1_Corynebacterium_glutamicum
WP_068430993.1_Piscicoccus_intestinalis
WP_083163319.1_Mycobacterium_aquaticum
WP_081714872.1_Helicobacter_fennelliae
EFP61298.1_Erysipelotrichaceae_bacterium
WP_081341564.1_Agrobacterium_vitis
ACB91984.1_Xylella_fastidiosa
WP_058671364.1_Enterobacter_hormaechei
XP_003293280.1_Dictyostelium_purpureum
WP_066288176.1_Arthrobacter_sp.
KFF47432.1_Pseudomonas_sp.
WP_003807566.1_Bordetella_bronchiseptica
WP_076327646.1_Paenibacillus_amylolyticus
SFN22858.1_Psychrobacillus_psychrodurans
WP_106028886.1_Bacillus_pumilus
WP_103372469.1_Staphylococcus_nepalensis
CDA62234.1_Clostridium_sp.
ORX88120.1_Anaeromyces_robustus
OUM60671.1_Piromyces_sp.
WP_049974915.1_Blautia_schinkii
SCJ78981.1_Eubacterium_sp.
ORY22739.1_Neocallimastix_californiae
WP_087218163.1_Blautia_sp.
PGH24004.1_Fusobacterium_nucleatum
WP_102834397.1_Clostridioides_difficile
OBZ33202.1_Megasphaera_sp.
WP_094636049.1_Bifidobacterium_eulemuris
CDA18180.1_Acetobacter_sp.
ERJ30873.1_Campylobacter_concisus
WP_082987518.1_Gallibacterium_genomosp.
KXB49252.1_Corynebacterium_kroppenstedtii
WP_009142612.1_Succinatimonas_hippei
WP_082942594.1_Helicobacter
CDD81106.1_Dialister_sp.
WP_006915872.1_Lactobacillus_coleohominis
WP_075888181.1_Lactobacillus_crustorum
WP_010788710.1_Bacillus_atrophaeus
OJG89486.1_Enterococcus_raffinosus
WP_009302400.1_Desulfovibrio_sp.
WP_049687050.1_Dakarella_massiliensis
SNX32375.1_Lactobacillus_fermentum
WP_018371447.1_Streptococcus_massiliensis
WP_002607579.1_Clostridium
WP_071861943.1_Enterococcus_devriesei
CEI82750.1_Oceanobacillus_oncorhynchi
WP_006555587.1_Veillonella_seminalis
0.4

E. 2,5-diketo-D-gluconic acid reductase 1

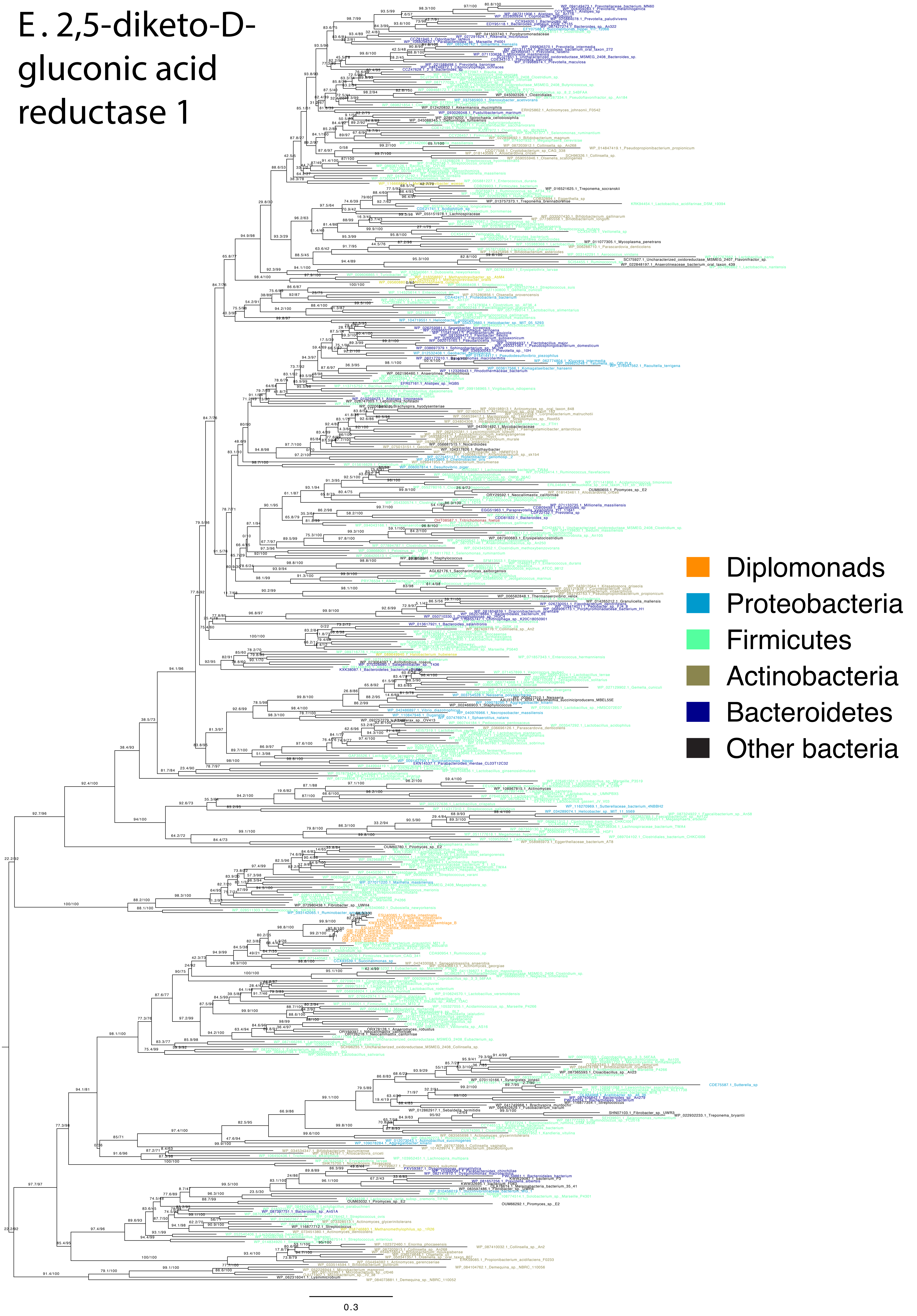

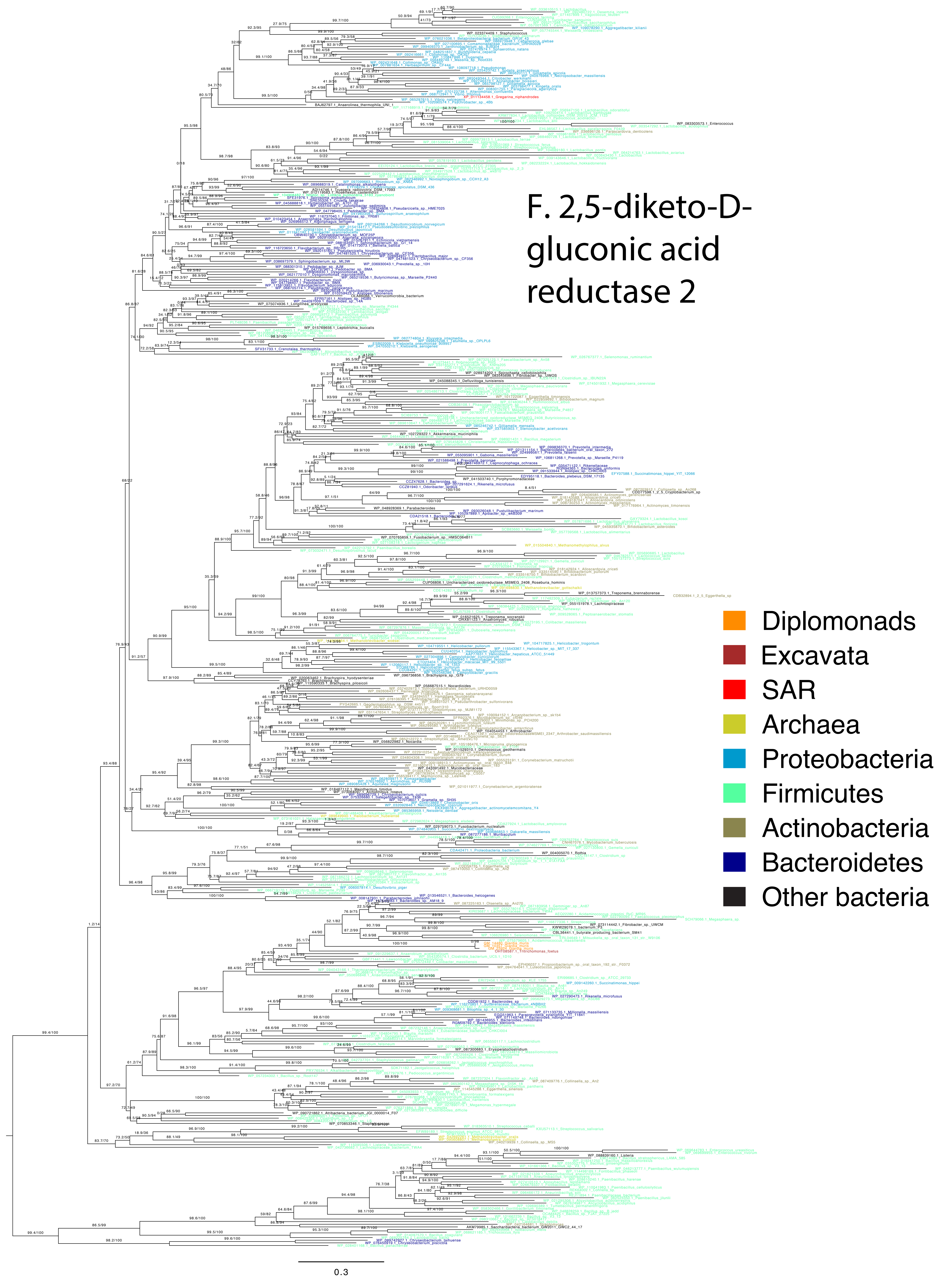
F. 2,5-diketo-D-gluconic acid reductase 2
Diplomonads
Excavata
SAR
Archaea
Proteobacteria
Firmicutes
Actinobacteria
Bacteroidetes
Other bacteria
0.3

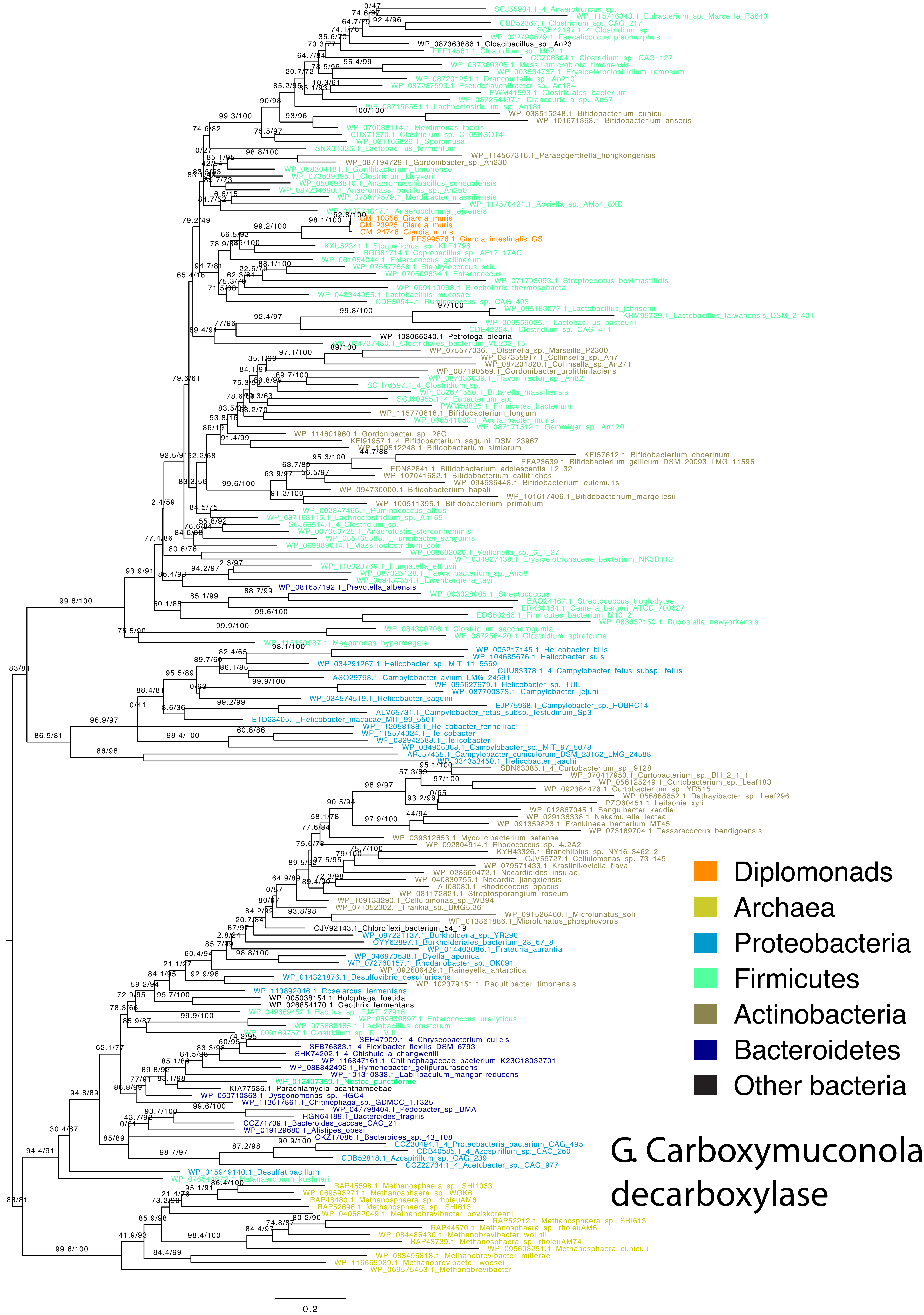

G. Carboxymuconolactone decarboxylase

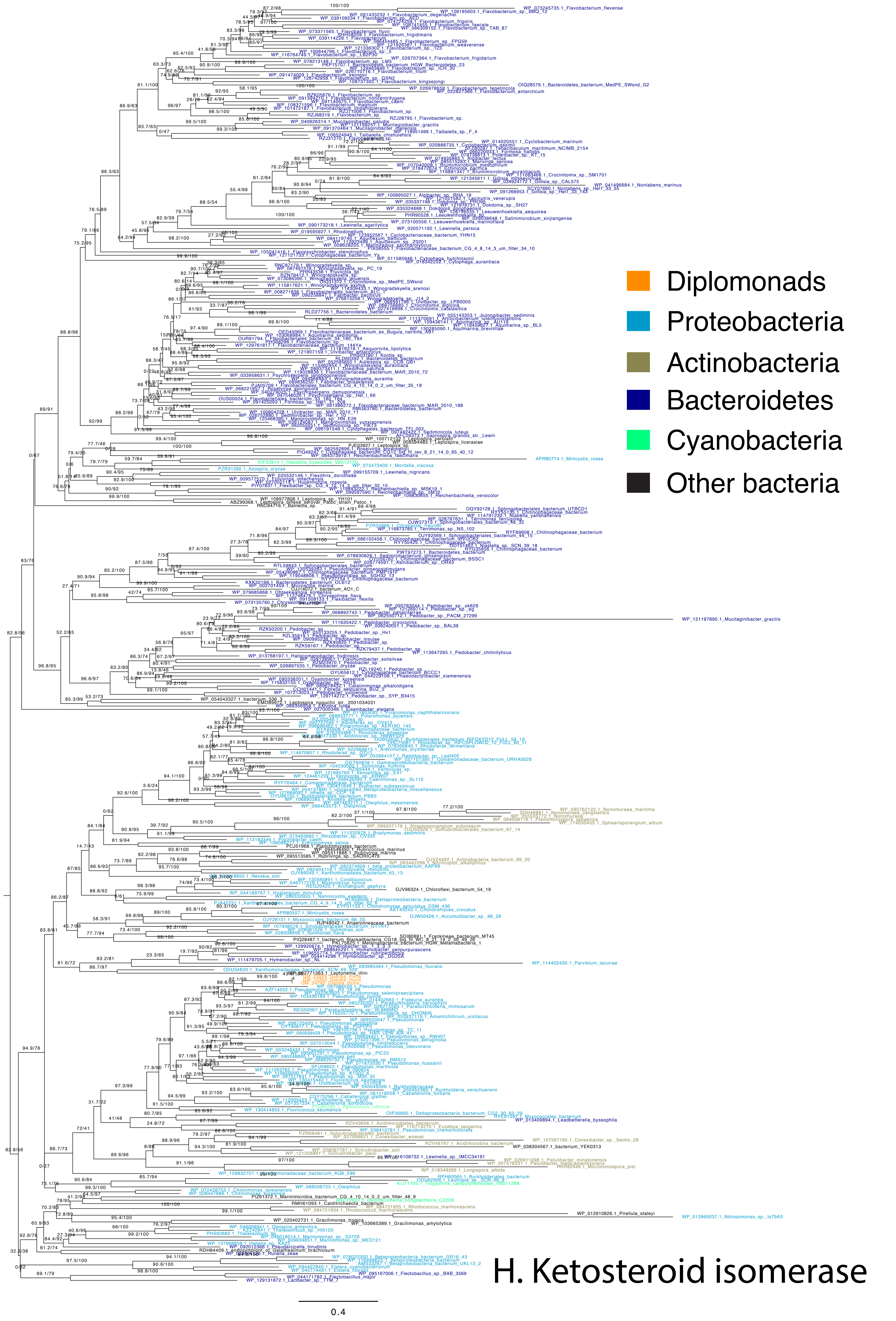
Diplomonads
Proteobacteria
Actinobacteria
Bacteroidetes
Cyanobacteria
Other bacteria
H. Ketosteroid isomerase
0.4

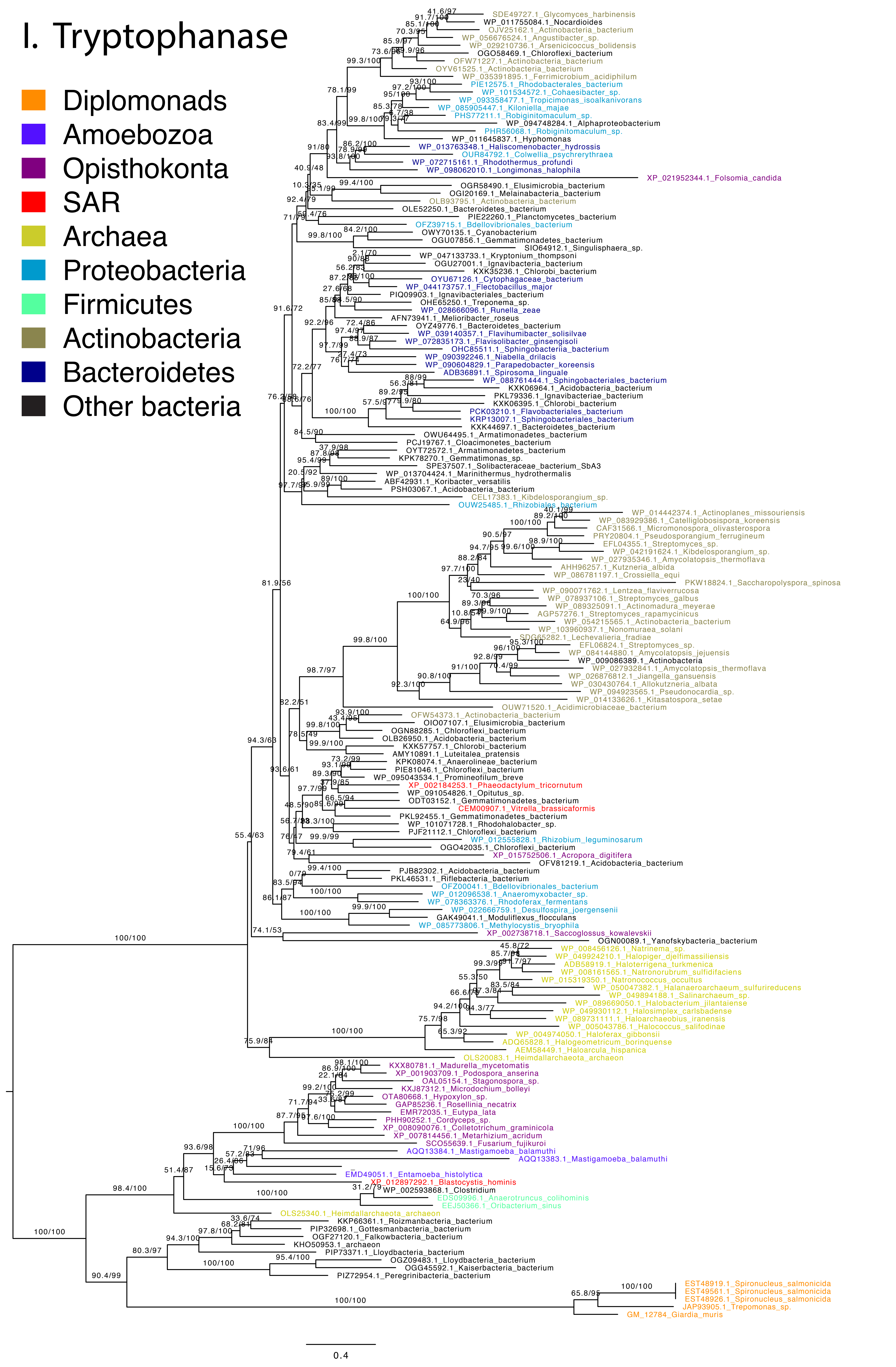
I. Tryptophanase
Diplomonads
Amoebozoa
Opisthokonta
SAR
Archaea
Proteobacteria
Firmicutes
Actinobacteria
Bacteroidetes
Other bacteria
SDE49727.1_Glycomyces_harbinensis
WP_011755084.1_Nocardioides
OJV25162.1_Actinobacteria_bacterium
WP_056676524.1_Angustibacter_sp.
WP_029210736.1_Arsenicicoccus_bolidensis
OGO58469.1_Chloroflexi_bacterium
OFW71227.1_Actinobacteria_bacterium
OYV61525.1_Actinobacteria_bacterium
WP_035391895.1_Ferrimicrobium_acidiphilum
PIE12575.1_Rhodobacterales_bacterium
WP_101534572.1_Cohaesibacter_sp.
WP_093358477.1_Tropicimonas_isoalkanivorans
WP_085905447.1_Kiloniella_majae
PHS77211.1_Robiginitomaculum_sp.
WP_094748284.1_Alphaproteobacterium
PHR56068.1_Robiginitomaculum_sp.
WP_011645837.1_Hyphomonas
WP_013763348.1_Haliscomenobacter_hydrossis
OUR84792.1_Colwellia_psychrerythraea
WP_072715161.1_Rhodothermus_profundi
WP_098062010.1_Longimonas_halophila
XP_021952344.1_Folsomia_candida
OGR58490.1_Elusimicrobia_bacterium
OGI20169.1_Melainabacteria_bacterium
OLB93795.1_Actinobacteria_bacterium
OLE52250.1_Bacteroidetes_bacterium
PIE22260.1_Planctomycetes_bacterium
OFZ39715.1_Bdellovibrionales_bacterium
OWY70135.1_Cyanobacterium
OGU07856.1_Gemmatimonadetes_bacterium
SIO64912.1_Singulisphaera_sp.
WP_047133733.1_Kryptonium_thompsoni
OGU27001.1_Ignavibacteria_bacterium
KXK35236.1_Chlorobi_bacterium
OYU67126.1_Cytophagaceae_bacterium
WP_044173757.1_Flectobacillus_major
PIQ09903.1_Ignavibacteriales_bacterium
OHE65250.1_Treponema_sp.
WP_028666096.1_Runella_zeae
AFN73941.1_Melioribacter_roseus
OYZ49776.1_Bacteroidetes_bacterium
WP_039140357.1_Flavihumibacter_solisilvae
WP_072835173.1_Flavisolibacter_ginsengisoli
OHC85511.1_Sphingobacteriia_bacterium
WP_090392246.1_Niabella_drilacis
WP_090604829.1_Parapedobacter_koreensis
ADB36891.1_Spirosoma_linguale
WP_088761444.1_Sphingobacteriales_bacterium
KXK06964.1_Acidobacteria_bacterium
PKL79336.1_Ignavibacteriae_bacterium
KXK06395.1_Chlorobi_bacterium
PCK03210.1_Flavobacteriales_bacterium
KRP13007.1_Sphingobacteriales_bacterium
KXK44697.1_Bacteroidetes_bacterium
OWU64495.1_Armatimonadetes_bacterium
PCJ19767.1_Cloacimonetes_bacterium
OYT72572.1_Armatimonadetes_bacterium
KPK78270.1_Gemmatimonas_sp.
SPE37507.1_Solibacteraceae_bacterium_SbA3
WP_013704424.1_Marinithermus_hydrothermalis
ABF42931.1_Koribacter_versatilis
PSH03067.1_Acidobacteria_bacterium
CEL17383.1_Kibdelosporangium_sp.
OUW25485.1_Rhizobiales_bacterium
WP_014442374.1_Actinoplanes_missouriensis
WP_083929386.1_Catelliglobosispora_koreensis
CAF31566.1_Micromonospora_olivasterospora
PRY20804.1_Pseudosporangium_ferrugineum
EFL04355.1_Streptomyces_sp.
WP_042191624.1_Kibdelosporangium_sp.
WP_027935346.1_Amycolatopsis_thermoflava
AHH96257.1_Kutzneria_albida
WP_086781197.1_Crossiella_equi
PKW18824.1_Saccharopolyspora_spinosa
WP_090071762.1_Lentzea_flaviverrucosa
WP_078937106.1_Streptomyces_galbus
WP_089325091.1_Actinomadura_meyerae
AGP57276.1_Streptomyces_rapamycinicus
WP_054215565.1_Actinobacteria_bacterium
WP_103960937.1_Nonomuraea_solani
SDG65282.1_Lechevalieria_fradiae
EFL06824.1_Streptomyces_sp.
WP_084144880.1_Amycolatopsis_jejuensis
WP_009086389.1_Actinobacteria
WP_027932841.1_Amycolatopsis_thermoflava
WP_026876812.1_Jiangella_gansuensis
WP_030430764.1_Allokutzneria_albata
WP_094923565.1_Pseudonocardia_sp.
WP_014133626.1_Kitasatospora_setae
OUW71520.1_Acidimicrobiaceae_bacterium
OFW54373.1_Actinobacteria_bacterium
OIO07107.1_Elusimicrobia_bacterium
OGN88285.1_Chloroflexi_bacterium
OLB26950.1_Acidobacteria_bacterium
KXK57757.1_Chlorobi_bacterium
AMY10891.1_Luteitalea_pratensis
KPK08074.1_Anaerolineae_bacterium
PIE81046.1_Chloroflexi_bacterium
WP_095043534.1_Promineofilum_breve
XP_002184253.1_Phaeodactylum_tricornutum
WP_091054826.1_Opitutus_sp.
ODT03152.1_Gemmatimonadetes_bacterium
CEM00907.1_Vitrella_brassicaformis
PKL92455.1_Gemmatimonadetes_bacterium
WP_101071728.1_Rhodohalobacter_sp.
PJF21112.1_Chloroflexi_bacterium
WP_012555828.1_Rhizobium_leguminosarum
OGO42035.1_Chloroflexi_bacterium
XP_015752506.1_Acropora_digitifera
OFV81219.1_Acidobacteria_bacterium
PJB82302.1_Acidobacteria_bacterium
PKL46531.1_Riflebacteria_bacterium
OFZ00041.1_Bdellovibrionales_bacterium
WP_012096538.1_Anaeromyxobacter_sp.
WP_078363376.1_Rhodoferax_fermentans
WP_022666759.1_Desulfospira_joergensenii
GAK49041.1_Moduliflexus_flocculans
WP_085773806.1_Methylocystis_bryophila
XP_002738718.1_Saccoglossus_kowalevskii
OGN00089.1_Yanofskybacteria_bacterium
WP_008458126.1_Natrinema_sp.
WP_049924210.1_Halopiger_djelfimassiliensis
ADB58919.1_Haloterrigena_turkmenica
WP_008161565.1_Natronorubrum_sulfidifaciens
WP_015319350.1_Natronococcus_occultus
WP_050047382.1_Halanaeroarchaeum_sulfurireducens
WP_049894188.1_Salinarchaeum_sp.
WP_089669050.1_Halobacterium_jilantaiense
WP_049930112.1_Halosimplex_carlsbadense
WP_089731111.1_Haloarchaeobius_iranensis
WP_005043786.1_Halococcus_salifodinae
WP_004974050.1_Haloferax_gibbonsii
ADQ65828.1_Halogeometricum_borinquense
AEM58449.1_Haloarcula_hispanica
OLS20083.1_Heimdallarchaeota_archaeon
KXX80781.1_Madurella_mycetomatis
XP_001903709.1_Podospora_anserina
OAL05154.1_Stagonospora_sp.
KXJ87312.1_Microdochium_bolleyi
OTA80668.1_Hypoxylon_sp.
GAP85236.1_Rosellinia_necatrix
EMR72035.1_Eutypa_lata
PHH90252.1_Cordyceps_sp.
XP_008090076.1_Colletotrichum_graminicola
XP_007814456.1_Metarhizium_acridum
SCO55639.1_Fusarium_fujikuroi
AQQ13384.1_Mastigamoeba_balamuthi
AQQ13383.1_Mastigamoeba_balamuthi
EMD49051.1_Entamoeba_histolytica
XP_012897292.1_Blastocystis_hominis
WP_002593868.1_Clostridium
EDS09996.1_Anaerotruncus_colihominis
EEJ50366.1_Oribacterium_sinus
OLS25340.1_Heimdallarchaeota_archaeon
KKP66361.1_Roizmanbacteria_bacterium
PIP32698.1_Gottesmanbacteria_bacterium
OGF27120.1_Falkowbacteria_bacterium
KHO50953.1_archaeon
PIP73371.1_Lloydbacteria_bacterium
OGZ09483.1_Lloydbacteria_bacterium
OGG45592.1_Kaiserbacteria_bacterium
PIZ72954.1_Peregrinibacteria_bacterium
EST48919.1_Spironucleus_salmonicida
EST49561.1_Spironucleus_salmonicida
EST48926.1_Spironucleus_salmonicida
JAP93905.1_Trepomonas_sp.
GM_12784_Giardia_muris
0.4

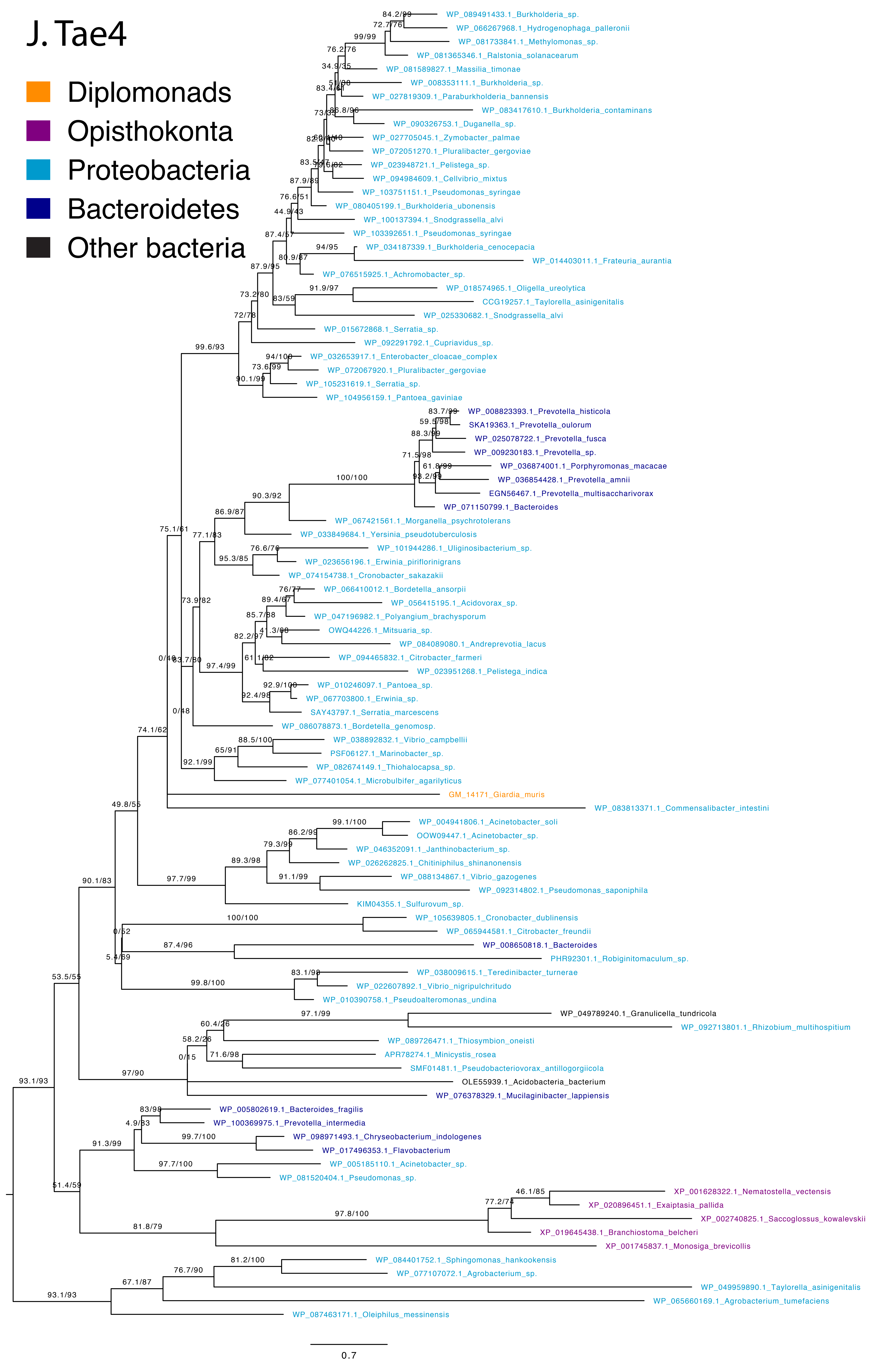
J. Tae4
Diplomonads
Opisthokonta
Proteobacteria
Bacteroidetes
Other bacteria
WP_089491433.1_Burkholderia_sp.
WP_066267968.1_Hydrogenophaga_palleronii
WP_081733841.1_Methylomonas_sp.
WP_081365346.1_Ralstonia_solanacearum
WP_081589827.1_Massilia_timonae
WP_008353111.1_Burkholderia_sp.
WP_027819309.1_Paraburkholderia_bannensis
WP_083417610.1_Burkholderia_contaminans
WP_090326753.1_Duganella_sp.
WP_027705045.1_Zymobacter_palmae
WP_072051270.1_Pluralibacter_gergoviae
WP_023948721.1_Pelistega_sp.
WP_094984609.1_Cellvibrio_mixtus
WP_103751151.1_Pseudomonas_syringae
WP_080405199.1_Burkholderia_ubonensis
WP_100137394.1_Snodgrassella_alvi
WP_103392651.1_Pseudomonas_syringae
WP_034187339.1_Burkholderia_cenocepacia
WP_014403011.1_Frateuria_aurantia
WP_076515925.1_Achromobacter_sp.
WP_018574965.1_Oligella_ureolytica
CCG19257.1_Taylorella_asinigenitalis
WP_025330682.1_Snodgrassella_alvi
WP_015672868.1_Serratia_sp.
WP_092291792.1_Cupriavidus_sp.
WP_032653917.1_Enterobacter_cloacae_complex
WP_072067920.1_Pluralibacter_gergoviae
WP_105231619.1_Serratia_sp.
WP_104956159.1_Pantoea_gaviniae
WP_008823393.1_Prevotella_histicola
SKA19363.1_Prevotella_oulorum
WP_025078722.1_Prevotella_fusca
WP_009230183.1_Prevotella_sp.
WP_036874001.1_Porphyromonas_macacae
WP_036854428.1_Prevotella_amnii
EGN56467.1_Prevotella_multisaccharivorax
WP_071150799.1_Bacteroides
WP_067421561.1_Morganella_psychrotolerans
WP_033849684.1_Yersinia_pseudotuberculosis
WP_101944286.1_Uliginosibacterium_sp.
WP_023656196.1_Erwinia_piriflorinigrans
WP_074154738.1_Cronobacter_sakazakii
WP_066410012.1_Bordetella_ansorpii
WP_056415195.1_Acidovorax_sp.
WP_047196982.1_Polyangium_brachysporum
OWQ44226.1_Mitsuaria_sp.
WP_084089080.1_Andreprevotia_lacus
WP_094465832.1_Citrobacter_farmeri
WP_023951268.1_Pelistega_indica
WP_010246097.1_Pantoea_sp.
WP_067703800.1_Erwinia_sp.
SAY43797.1_Serratia_marcescens
WP_086078873.1_Bordetella_genomosp.
WP_038892832.1_Vibrio_campbellii
PSF06127.1_Marinobacter_sp.
WP_082674149.1_Thiohalocapsa_sp.
WP_077401054.1_Microbulbifer_agarilyticus
GM_14171_Giardia_muris
WP_083813371.1_Commensalibacter_intestini
WP_004941806.1_Acinetobacter_soli
OOW09447.1_Acinetobacter_sp.
WP_046352091.1_Janthinobacterium_sp.
WP_026262825.1_Chitiniphilus_shinanonensis
WP_088134867.1_Vibrio_gazogenes
WP_092314802.1_Pseudomonas_saponiphila
KIM04355.1_Sulfurovum_sp.
WP_105639805.1_Cronobacter_dublinensis
WP_065944581.1_Citrobacter_freundii
WP_008650818.1_Bacteroides
PHR92301.1_Robiginitomaculum_sp.
WP_038009615.1_Teredinibacter_turnerae
WP_022607892.1_Vibrio_nigripulchritudo
WP_010390758.1_Pseudoalteromonas_undina
WP_049789240.1_Granulicella_tundricola
WP_092713801.1_Rhizobium_multihospitium
WP_089726471.1_Thiosymbion_oneisti
APR78274.1_Minicystis_rosea
SMF01481.1_Pseudobacteriovorax_antillogorgiicola
OLE55939.1_Acidobacteria_bacterium
WP_076378329.1_Mucilaginibacter_lappiensis
WP_005802619.1_Bacteroides_fragilis
WP_100369975.1_Prevotella_intermedia
WP_098971493.1_Chryseobacterium_indologenes
WP_017496353.1_Flavobacterium
WP_005185110.1_Acinetobacter_sp.
WP_081520404.1_Pseudomonas_sp.
XP_001628322.1_Nematostella_vectensis
XP_020896451.1_Exaiptasia_pallida
XP_002740825.1_Saccoglossus_kowalevskii
XP_019645438.1_Branchiostoma_belcheri
XP_001745837.1_Monosiga_brevicollis
WP_084401752.1_Sphingomonas_hankookensis
WP_077107072.1_Agrobacterium_sp.
WP_049959890.1_Taylorella_asinigenitalis
WP_065660169.1_Agrobacterium_tumefaciens
WP_087463171.1_Oleiphilus_messinensis
0.7

K. Quorum-quenching N-acyl-homoserine lactonase

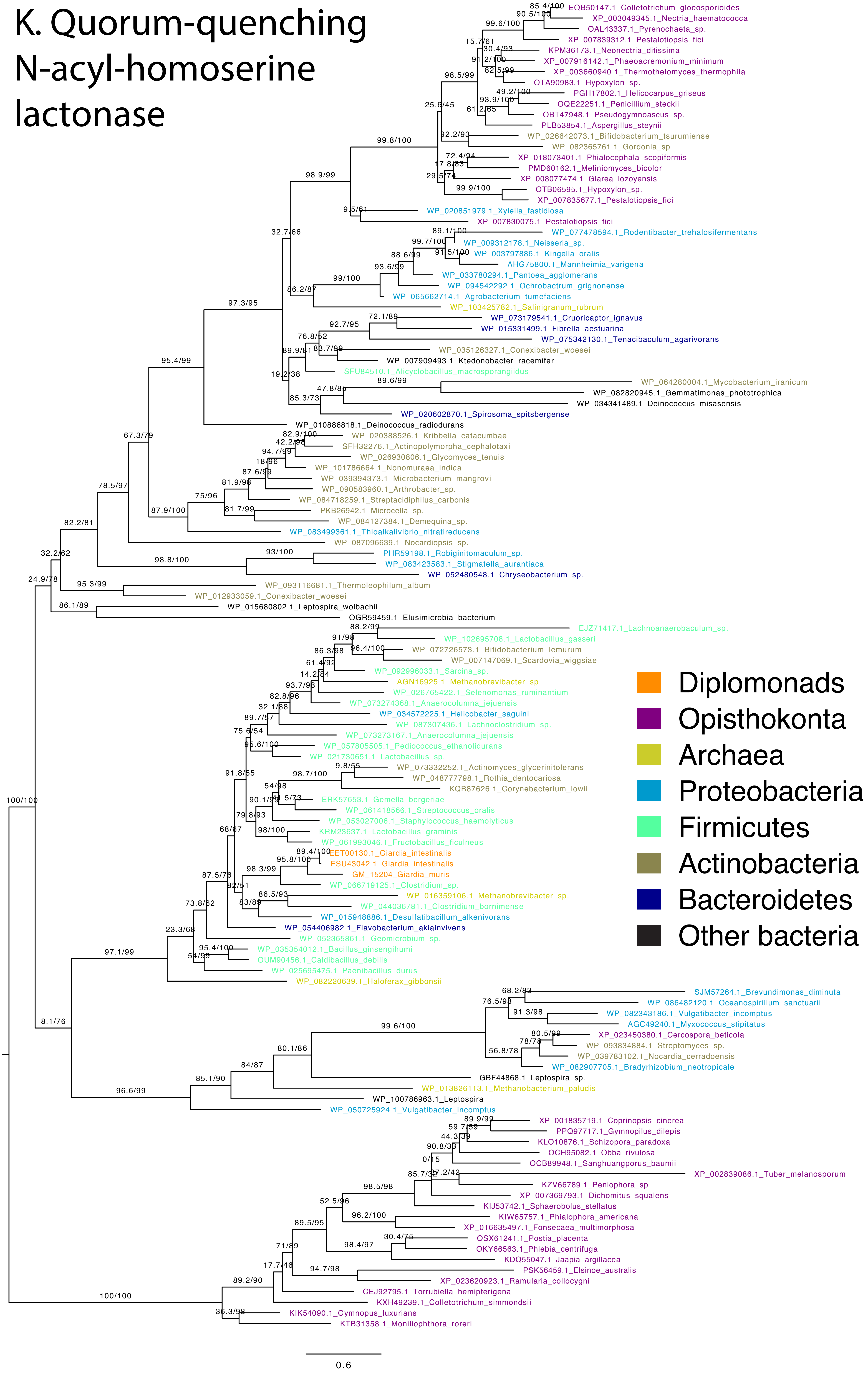

Methods S1 – Compact genome of *Giardia muris* reveals important steps in the evolution of intestinal protozoan parasites” by Xu et al. (2020)

DNA preparation from *G. muris* for long read sequencing

G. muris excyzoites (~9×10^7 cells) were centrifuged at 500 ×g for five minutes to remove the excystation media and the pellet was dissolved in Lysis Buffer (50 mM EDTA, 1% SDS and 10 mg/ml Proteinase K) by vortexing. The lysed cells were incubated at 56 °C with occasional vortexing for 2 hours followed by addition of 1 µl RNase A (100 mg/µl) and incubation at room temperature for 10-15 minutes. The genomic DNA was extracted by addition of 0.5 volumes each of phenol and CHISAM (chloroform: isoamyl alcohol 24:1). The sample was mixed carefully by vortexing and centrifuged at 13,000 rpm for 10 minutes, the water phase was collected and an equal volume of CHISAM was added to the sample and mixed by vortexing followed by a second centrifugation with same settings. The water phase was collected, and the DNA was precipitated with an equal volume of isopropanol. The precipitate was recovered by immediate centrifugation at 13,000 rpm, 4 °C, 30 min. The supernatant was removed, and the pellet was washed with 70% ice cold ethanol and then centrifuged at 13,000 rpm, 4 °C, 10 minutes. The supernatant was removed, air-dried briefly and resuspended in TE-buffer (10 mM Tris-HCl pH 8.0, 1 mM EDTA). The genomic DNA was further purified using Genomic-tip 20/G (Qiagen) according to the manufacturers instructions.

Finally, the genomic DNA was purified using phenol-chloroform extraction according to the “Extracting DNA Using Phenol-Chloroform” protocol (https://www.pacb.com/wp-content/uploads/2015/09/Experimental-Protocol-Extracting-DNA-using-Phenol-Chloroform.pdf). The DNA solution was mixed with an equal volume of

the phenol/chloroform/isoamyl alcohol solution (25:24:1). The tube was mixed vigorously for 1 min and spun at high speed for 5 min. The aqueous phase was removed, and the tube was back extracted using EB buffer. The original and back-extracted material was combined and extracted as above using chloroform/isoamyl alcohol (24:1). The aqueous phase was precipitated using 2.5× volume 100% ethanol and NH_4OAc (0.75 M final concentration) while adding glycogen (20 ug /extraction). The precipitate was recovered by high speed centrifugation and washed two times by double volumes of 80% ethanol. The pellet was air-dried and brought up in a small volume of EB buffer. The concentration and quality of the extracted DNA was determined by NanoDrop and agarose gel electrophoresis.

RNA purification

Total RNA from trophozoites were extracted using TriZol according to the method in Franzén et al.[1]. The total RNA was DNase treated using the Turbo DNA-free kit (Applied Biosystems, AM1907) as described in Franzén et al.[1]. RNA from cysts and excyzoites were extracted using TriZol as described above and the resulting RNA was DNase treated (Turbo DNase, 2 U/µl) in a reaction volume of 100 µl for 30 minutes at 37°C. The RNA was subsequently cleaned using the cleanup protocol from the RNeasy Mini Kit (Qiagen, 74104) and eluted in 30 µl of DEPC treated water. The quality and quantity were evaluated using Qubit and BioAnalyzer.

Sequencing

Total genomic DNA was sequenced using both Illumina MiSeq and PacBio RS II sequencers at the SNP&SEQ platform and Uppsala Genome Center respectively at the Science for Life Laboratory (Uppsala University). The trophozoite total RNA was divided in two parts and stranded transcriptome mRNA library was prepared using the appropriate TruSeq kit at GATC Biotech (Konstanz, Germany). The finished libraries were sequenced using the Illumina HiSeq 2000 system at GATC Biotech (Konstanz, Germany). RNA sequencing

libraries from cysts and excyzoites were prepared using the TruSeq stranded mRNA sample preparation kit and sequenced by HiSeq 2500 at the SNP&SEQ platform.

Raw DNA and RNA sequence reads are archived at NCBI Sequence Read Archive (SRA) under accession numbers SRR8858297-SRR8858305.

Biosamples and accession numbers

**Biosample accession**	**SAMN11231833**		**SAMN11231832**		**SAMN11231834**
**Sample description**	Cysts		Trophozoites		Excysted trophozoites
**DNA/RNA**	DNA	RNA	DNA	RNA	RNA
**Sequencing instrument**	PacBio RS II	Illumina HiSeq 2500	Illumina MiSeq	Illumina HiSeq 2000	Illumina HiSeq 2500
**Usage**	Genome assembly	RNA-Seq comparison	Assembly base correction	RNA-Seq comparison	RNA-Seq comparison
**SRA accession**	SRR8858302-SRR8858305	SRR8858298	SRR8858300	SRR8858301	SRR8858299
**# Reads**	200,601	31,397,561	38,048,422	121,271,107	30,381,809
**Read len (bp)**	-	125	251*2	51	125
**# Total Bbp**	2.5	3.9	19.1	6.2	3.8

Repeat detection

RepeatMasker v3.3.0 (http://www.repeatmasker.org/) was run to detect genome repeats. Sequence comparison in RepeatMasker was performed by cross_match v1.080812. RM database v20110920 with RepBase Update 20110920 was used as RepeatMasker library.

Heterozygosity estimation

Samtools mpileup with B flag was used to generate pileup file from MiSeq reads mapped bam file. Sites with at least 20X coverage and 10% of alternative bases were considered as allelic heterozygous sites.

Genome annotation

Two gene prediction programs were used, Prodigal v2.60 and GlimmerHMM v3.0.1. Training set for GlimmerHMM consists of genes predicted from Prodigal which have hits against *G. intestinalis* with evalue <1e-50 and similar length to the hit gene. The union of the two sets of predicted genes was taken for functional annotation. Genes were manually annotated using information from BLASTP results against NR database as well as HMMER (v3.0) search results of domain information against Pfam (v27.0).

tRNA and rRNA

18S, 28S and 5.8S ribosomal RNAs were annotated after BLASTN similarity to the deposited *G. muris* ribosomal RNAs in NCBI (X65063), and the start and stop positions were adjusted to be inline with the record. 5S rRNAs were predicted by searching against Rfam 11.0 using infernal v1.0.2. There are 78 pieces of 28S rRNAs, 73 18S rRNAs, 68 5.8S rRNAs and 10 5S rRNA annotated.

tRNAs were predicted by tRNAScan-SE v1.23 with the most sensitive co-variance model. BLASTN with -task blastn (which is more sensitive for distant matches) of the 40 *G. intestinalis* ncRNAs against *G. muris* genome revealed 10 ncRNAs with evalue <0.1 and not overlapping genes. Alignment of the 10 ncRNAs using MUSCLE v3.8.31 showed a clear motif on the 3' end, CCTTYNHTNAA, which is similar to what was found on *G. intestinalis* (CCTTYNHTHAA). This motif was used to search for other potential ncRNAs in *G. muris* using scan_for_matches (http://blog.theseed.org/servers/2010/07/scan-for-matches.html). Additionally, results were obtained from BLASTN search against miRBase, similarity search against Rfam and prediction of small RNAs based on small RNA-Seq data using Shortstack v1.2.4. All the search and prediction results were combined following the rule that ncRNAs do not overlap (or overlap little) with the annotated genes and with themselves. Manual efforts were also applied. In the end, they are 219 ncRNAs annotated, with 16 from similarity

search against *G. intestinalis* ncRNAs, 8 from Rfam searches, 17 from Shortstack prediction, and 178 from motif search.

Protein kinases

Protein kinases were assigned into group, family and subfamily. Alignment files for different group, family and subfamily were downloaded from the kinase database (http://kinase.com). HMMER 3.0 (http://hmmer.janelia.org) was used to build HMM profiles from the alignment and the protein sequences were searched against the HMM profiles. The search results were then combined with Pfam domain search results where genes contain significant Pfam Pkinase domain (PF00069) (score >25). *G. intestinalis* protein kinase annotations were taken from reference.

Ankyrin repeat proteins

Ankyrin repeat proteins (ARPs) were assigned to genes that contain ankyrin repeat domains (score >=20) but not Pkinase domain. According to if an ARP contains zink finger domains or other domains, they were further divided into 3 subgroups.

Cysteine-rich proteins

Alignments of the proteins with more than 10% cysteines using MAFFT v7.215 revealed that a subset of them contains a conserved pentapeptide motif (GR)CR(GRKE)K at the C-terminus. Cysteine-rich proteins were divided into three subgroups depending on the presence and absence of the transmembrane domains and the pentapeptide motif.

Synteny

MUMmer v3.23 was used to align the draft genomes of *G. muris* and *G. intestinalis*. Promer was used in order to align the genome at protein level. Mummerplot was used to generate the alignment dot plot. Show-coords was used to view a summary of all the alignments produced by promer. Alignment coordinates were used to calculate the percentage of similarity between the two genomes as well as to draw the similarity in Circos plot.

OrthoMCL v2.0.2 was used to cluster genes using match cutoff of 40% and e-value cutoff of 1e-10. The clustering was done within *G. muris*, between *G. muris* and *G. intestinalis*, and among *G. muris*, *G. intestinalis* and *S. salmonicida*.

Phylogenetic trees for NEKs and ARPs

All the NEKs from *G. muris* and *G. intestinalis* were aligned with MAFFT v7.407 (auto setting), and FastTree v2.1.10 No SSE3 was used to construct approximately-maximum-likelihood tree using default WAG+CAT model. ARPs tree was constructed the same way as NEKs tree.

Phylogenetic analysis of lateral transfers

One sequence from *G. muris* was used as a query for each enzyme to retrieve at least 5000 hits with e-value <0.001, using BLASTP against the nr database and the organism-specific proteomes. The resulting datasets were filtered automatically by finding all sequences with greater than 90% sequence identity to another sequence using the software skipredundant (EMBOSS v6.5.7.0 [2]) with default settings. Datasets were aligned using MAFFT v6.603b[3], and regions of ambiguous alignment were automatically trimmed using BMGE v1.12 (blosum30 and block size of 2) [4]. Sequences with a length less than 50% of the multiple alignment were removed, and the datasets were aligned and trimmed again as described above. An initial phylogenetic analyses were performed using FastTree v2.1.8 SSE3, OpenMP [5]. Sequences with a phylogenetic distance < 0.3 were removed in an iterative process to create the final datasets. This final datasets were aligned in forward and reverse orientation using MAFFT and PROBCONS v1.12 [6]. The four resulting alignments were combined with T-COFFEE v11.00.8cbe486 [7] into a maximal consensus alignment, which was trimmed using BMGE as in previous steps. Maximum Likelihood (ML) trees were computed using IQtree v1.6.5 [8] under LG4X substitution model [9]. Branch supports were

assessed using ultrafast bootstrap approximation (UFboot) with 1,000 ultrafast bootstraps [10] and 1,000 replicates for SH-like approximate likelihood ratio test (SH-aLRT) [11].

Promoter motif search

MEME suite v4.10.2 was used for promoter motif analysis. A maximum of 120 bp upstream of annotated genes were used in search of potential promoter. The longest possible sequence was used if the intergenic region is shorter than 120 bp, and the sequence was discarded if the intergenic region is shorter than 8 bp. 4139 sequences in total were used in motif search, and MEME was set to search for 10 most likely motifs with sizes from 6 to 30 bp.

VSP and ψVSP phylogenetic analysis

Nucleotide sequences of *G. muris* VSPs and ψVSP genes were aligned using MAFFT in MAFFT v7.416[3], and regions of ambiguity were trimmed using BMGE v1.12 (DNAPAM100:2, [4]). Maximum Likelihood (ML) trees were computed using IQtree v.1.6.5 [8] under the best model (TIM2+F+R6) with 1,000 ultrafast bootstraps [10] and 1,000 bootstrap replicates for SH-like approximate likelihood ratio test (SH-aLRT)[11].

References

1. Franzén O, Jerlström-Hultqvist J, Einarsson E, Ankarklev J, Ferella M, Andersson B, et al. Transcriptome Profiling of Giardia intestinalis Using Strand-specific RNA-Seq. PLoS Computational Biology. 2013; 9(3):e1003000. doi:10.1371/journal.pcbi.1003000.

2. Rice P, Longden I, Bleasby A. EMBOSS: The European Molecular Biology Open Software Suite. Trends in Genetics. 2000; 16(1):276–277. doi:10.1016/j.cocis.2008.07.002.

3. Katoh K, Misawa K, Kuma K, Miyata T. MAFFT: a novel method for rapid multiple sequence alignment based on fast Fourier transform. Nucleic acids research. 2002; 30(14):3059–3066. doi:10.1093/nar/gkf436.

4. Criscuolo A, Gribaldo S. BMGE (Block Mapping and Gathering with Entropy): a new software for selection of phylogenetic informative regions from multiple sequence alignments. BMC evolutionary biology. 2010; 10(1):210. doi:10.1186/1471-2148-10-210.

5. Price MN, Dehal PS, Arkin AP. FastTree 2--approximately maximum-likelihood trees for large alignments. PLoS ONE. 2010; 5(3):e9490. doi:10.1371/journal.pone.0009490.

6. Do CB, Mahabhashyam MSP, Brudno M, Batzoglou S. ProbCons: Probabilistic consistency-based multiple sequence alignment. Genome research. 2005; 15(2):330–40. doi:10.1101/gr.2821705.

7. Notredame C, Higgins DG, Heringa J. T-Coffee: A novel method for fast and accurate multiple sequence alignment. J. Mol. Biol. 2000; 302(1):205–217. doi:10.1006/jmbi.2000.4042.

8. Nguyen L-T, Schmidt HA, von Haeseler A, Minh BQ. IQ-TREE: a fast and effective stochastic algorithm for estimating maximum-likelihood phylogenies. Mol. Biol. Evol. 2015; 32(1):268–274. doi:10.1093/molbev/msu300.

9. Kalyaanamoorthy S, Minh BQ, Wong TKF, von Haeseler A, Jermiin LS. ModelFinder: fast model selection for accurate phylogenetic estimates. Nat. Methods. 2017; 14(6):587–589. doi:10.1038/nmeth.4285.

10. Hoang DT, Chernomor O, von Haeseler A, Minh BQ, Vinh LS. UFBoot2: Improving the Ultrafast Bootstrap Approximation. Mol. Biol. Evol. 2018; 35(2):518–522. doi:10.1093/molbev/msx281.

11. Guindon S, Dufayard J-F, Lefort V, Anisimova M, Hordijk W, Gascuel O. New algorithms and methods to estimate maximum-likelihood phylogenies: assessing the performance of PhyML 3.0. Syst. Biol. 2010; 59(3):307–321. doi:10.1093/sysbio/syq010.
